# Emotions of subject and object affect beauty differently for images and music

**DOI:** 10.1167/jov.23.13.6

**Published:** 2023-11-16

**Authors:** Anna Bruns, Maria Pombo, Pablo Ripollés, Denis G. Pelli

**Affiliations:** 1 Center for Experimental Humanities, New York University, New York, NY, USA; 2 Department of Psychology, New York University, New York, NY, USA; 3 Music and Audio Research Lab (MARL), New York University, New York, NY, USA; 4 Center for Language, Music and Emotion (CLaME), New York University, Max-Planck Institute, New York, NY, USA; 5 Center for Neural Science, New York University, New York, NY, USA

**Keywords:** beauty, emotion, object emotion, mood, aesthetics, duration, art, music

## Abstract

What role do the emotions of subject and object play in judging the beauty of images and music? Eighty-one participants rated perceived beauty, liking, perceived happiness, and perceived sadness of 24 songs, 12 art images, and 12 nature photographs. Stimulus presentation was brief (2 seconds) or prolonged (20 seconds). The stimuli were presented in two blocks, and participants took the Positive and Negative Affect Score (PANAS) mood questionnaire before and after each block. They viewed a mood induction video between blocks either to increase their happiness or sadness or to maintain their mood. Using linear mixed-effects models, we found that perceived object happiness predicts an increase in image and song beauty regardless of duration. The effect of perceived object sadness on beauty, however, is stronger for songs than images and stronger for prolonged than brief durations. Subject emotion affects brief song beauty minimally and prolonged song beauty substantially. Whereas past studies of beauty and emotion emphasized sad music, here we analyze both happiness and sadness, both subject and object emotion, and both images and music. We conclude that the interactions between emotion and beauty are different for images and music and are strongly moderated by duration.

## Introduction

You can feel sad or you can recognize that a song is sad. Do these two kinds of sadness affect the beauty of the song? Although many aspects of aesthetic experience remain contested after centuries of debate, scholars and laypeople generally acknowledge that emotions play some role in it (Menninghaus et al., 2019; Schindler et al., 2017). First glimpse of the Grand Canyon or the Taj Mahal, the opening chords of “Clair de Lune,” and the well-chosen words of Pablo Neruda all seem to have an emotional power over the subjects who behold them. Beyond arousing emotion in subjects, such objects—regardless of their sensory modality—are often perceived as having distinct emotions as qualities, although these emotions are de-personified or distanced from mind and body. A listener can consider Chopin's “Nocturne No. 20” to be a “sad” song, even if it does not make them feel sad. Several studies explore this distinction between “felt” and “perceived” emotion in images and music (Evans & Schubert, 2008; Gabrielsson, 2001). Philosopher Stephen Davies (1980) elucidates the kinds of emotion one might perceive in music. Davies describes the emotion participants recognize in a stimulus as “emotion characteristics in appearance,” where what we mean when we say “that song is sad” is like what we mean when we say “that person is sad-looking.” When we say a person is sad looking, we do not mean the person's sad appearance is making us sad or even that we know the person currently feels sad, just that the person's appearance bears a sad quality. Similarly, a song could be called “sad” if it bears a sad quality, even if it does not make us sad, even if we do not assume its composer or performer was sad while creating it, and despite our belief that songs cannot literally feel sadness. In this study, we are interested in both the perceived emotions a subject recognizes as qualities of images and music as well as the felt emotions a subject might experience before, during, or after an encounter with these stimuli.

Some of the felt emotions that psychologists have studied in relation to images and music are those referred to as “aesthetic emotions,” emotions like awe and wonder that are associated with “aesthetic events” more than with “everyday events” (e.g., Leder & Nadal, 2014; Juslin, 2013; Menninghaus et al., 2019; Menninghaus et al., 2017). Yet the felt (and perceived) emotions of interest in the present study are those that some emotion theorists call “basic” or “everyday” emotions: happiness and sadness (Starr, 2013). And we are interested in both their felt and their perceived forms across two modalities: visual and auditory. We chose these modalities because they dominate aesthetics discourse.

This paper uses the term “object emotion” as a shorthand to refer to the emotion one might perceive in a stimulus and the term “subject emotion” to refer to the present emotional state of the person engaging with the stimulus. We want to unveil the relationship between these two kinds of emotions and beauty, after having seen results from related pilot data that seemed to conflict. Denis Pelli collected data showing no effect of subject emotion on the beauty of images, and Anna Bruns collected data showing strong effects of object emotion on the beauty of images and music. So perhaps object emotion affects beauty but subject emotion does not. But might these two kinds of emotion interact in their effect on beauty? Are the effects different for images versus music? How does emotional valence play a role?

One might assume that stimuli that arouse more pleasant and positively valenced emotions would be judged as more beautiful because beauty is widely linked with pleasure (Brielmann & Pelli, 2019; Kant, 2000). The Hagtvedt, Patrick, and Hagtvedt (2008) model for the perception and evaluation of visual art relies on the idea that “the influence of affect is typically congruent with its valence, such that a positive feeling leads to a positive evaluation while a negative feeling gives rise to a negative evaluation.” Yet, although some investigators claim that this congruence is typical, it is easy to name songs or visual artworks that evoke sadness (perceived or felt) and are judged by many as beautiful and pleasant to behold. Similarly, there is a thriving business of horror movies that focus on producing fear, to viewers’ delight. And surely it is possible to appreciate a stimulus as beautiful or otherwise experience an aesthetic event, pleasure and all, while in a sad emotional state. The former phenomenon—that sad music is often judged as very beautiful and pleasant—has inspired many scholarly projects within music theory, philosophy, psychology, and neuroscience (e.g., Killgore & Yurgelun-Todd, 2004; Sachs, Damasio, & Habibi, 2015; Surguladze et al., 2005; Vuoskoski & Eerola, 2017).

### Beauty and emotion of music

Eerola, Vuoskoski, Peltola, Putkinen, and Schäfer (2018) stress the diversity of emotions provoked by musical sadness. This sadness can be characterized by a range of experiences, including intensely pleasurable experiences, low arousal experiences like relaxation, and negative experiences like grief. Both relaxation and intense pleasure have intrigued and even mystified scholars: How can we enjoy sad music? Eerola et al. (2018) describe several studies that invoke music's lack of real-world consequences as a central factor enabling our enjoyment of sad music. For example, according to the Menninghaus et al. (2017) Distancing-Embracing model, the cognitive schemas of fiction associated with art distance the subject from the object and give the subject an assurance of personal control and physical safety. This allows the subject to embrace the sadness in the art object, which is then perceived to be “more intense, more interesting, more emotionally moving, more profound, and occasionally even more beautiful.”

However, in Barrett, Schulkin, and Bernacer's (2017) commentary on the Menninghaus et al. (2017) model, they criticize the “distancing” conjecture, saying sadness in music can be enjoyable in itself; no “hedonic flip” from painful feelings to pleasant ones is needed for enjoyment or aesthetic appreciation of sad music. Although Barrett et al. (2017) acknowledge that “enjoyable sadness seems oxymoronic,” they point out that “there is no evidence from self-report studies that subjects find musically induced feelings of sadness to be unpleasant in themselves and, thus, in need of ‘distancing’” (e.g., Taruffi & Koelsch, 2014; Vuoskoski, Thompson, McIlwain, & Eerola, 2012). Zatorre (2005) finds that sad music activates brain regions also associated with processing food, sex, and attachment and suggests that the pleasure experienced in response to sad music need not involve a hedonic shift. Pelowski, Ishizu, and Leder (2018) agree, proposing that, rather than a shift from an unpleasant to a pleasant experience, the pleasure of sad music might be enabled by a “shift from subjectively felt to more intellectually or cognitively perceived emotion.” A study by Kawakami, Furukawa, Katahira, and Okanoya (2013) supports this theory. They asked participants to rate songs in terms of the extent to which they perceived a series of emotions as qualities of the songs and to report their own felt emotions while listening. They found participants’ felt emotions to be “more romantic, more blithe, and less tragic” than the perceived emotions in the music, suggesting that the sadness in music might not match the felt emotion in listeners.

All of these characterizations of the beauty and enjoyment of sad music contribute to the field's understanding of how and why sad music is enjoyed. However, they rely on the primarily anecdote-backed assumption that sad music is generally enjoyed without verifying this assumption with empirical evidence. Is sad music generally enjoyed more than less sad music is? More than happy music is? The present study seeks answers to both questions.

### Beauty and emotion of pictures

Many of the theories described, like the Distancing-Embracing model and the theories that oppose it, apply to visual art in addition to music. In one project focused on images, Silvia (2005) argues against Berlyne's (1971) arousal theories of aesthetic response. Instead, Silvia favors appraisal theories that integrate emotion psychology. Silvia describes a study wherein participants rated their interest in a series of pictures and their ability to understand them, finding that “ability to understand” only predicted interest for complex pictures and not for simple ones. Berlyne took arousal to be the aesthetic response to the stimulus, and he studied the relationship between arousal and “collative properties” of art (complexity, novelty, incongruity, surprisingness). Silvia argues that arousal is insufficient to explain aesthetic response and that appraisal theories of emotion help complete the picture. Silvia claims that a complete theory of aesthetic response must account for everyday emotions. The present paper is a step in that direction.

A neuroscience study by Ishizu and Zeki (2017) applies appraisal theories of emotional response to aesthetic experience. They wanted to understand the neural mechanisms involved in aesthetic response to images that strongly evoked joy or sadness. They found that images evoking joy elicited greater activity in the right temporoparietal junction and those evoking sadness elicited greater activity in the left inferior parietal lobe. However, they found that both types of images elicited activity in the medial orbito-frontal cortex, a region Ishizu and Zeki (2011) had previously identified as a neural correlate of beauty. Ishizu and Zeki's (2017) study identifies brain regions associated with the intersection between aesthetic experience and everyday emotions. The present study has a similar goal—to explore the link between beauty and everyday emotions empirically—but it relies on behavioral methods rather than neuroscientific ones, and it looks at emotion as input (rather than output) to aesthetic experience.

### Subject emotion as an input to aesthetic response

Studies that look at the relationship between beauty and participants’ prior emotional state mostly deal with empathy (Eerola et al., 2018; Freedberg & Gallese, 2007; Gernot, Pelowski, & Leder, 2018; Wilkinson, Cunningham, & Elliott, 2021). Individual differences can affect the relevance of emotion to aesthetic experience, with highly empathic individuals showing higher levels of interest and emotional engagement in visual art (Wilkinson et al., 2021). Gernot et al. (2018) suggest that this heightened interest associated with emotion contagion—“the ability to pick up and mirror, or in short to ‘feel into’, emotions, which often overlaps with higher general or interpersonal empathetic abilities”—might help to explain our enjoyment of sad music. Previous research has also shown individual differences in sensitivity to reward, which conceivably could also modulate the influence of subject emotion on beauty. These differences can be measured by self-reports such as the Aesthetic Responsiveness Assessment test (AReA) (Schlotz et al., 2021) and the Barcelona Music Reward Questionnaire (BMRQ) (Mas-Herrero, Marco-Pallares, Lorenzo-Seva, Zatorre, & Rodriguez-Fornells, 2013), which we include in the present study.

Other theories of sad music enjoyment suppose a role for the music listener's emotional state. Sachs et al. (2015) propose that sad music restores homeostatic balance to a person in distress. In contrast, Taruffi and Koelsch (2014) find that people like sad music more when they are in a sad emotional state than when they are in a happy one, concluding that people prefer to listen to music congruent with their mood. However, the methods they used to draw these conclusions consisted only of questionnaires wherein participants provided 7-point Likert scale ratings in response to the statement, “When I am in a sad mood I like to listen to sad music” and “When I am in a positive mood I like to listen to sad music.” These tantalizing results are a natural lead-in to our investigation of the way subject and object emotions affect the beauty of images and music.

Furthermore, their study, like most empirical aesthetics studies, makes no direct comparison between effects for stimuli of different sensory modalities. Yet a self-report study by Miu, Pițur, and Szentágotai-Tătar (2016) gives hints that these differences may be worth studying. They asked participants about their engagement with paintings and music, finding that the most commonly mentioned contributors to emotional response to paintings were stimulus features and previous knowledge, whereas for music these were prior mood, physical context, and the presence of other people. This finding suggests that the relationship between emotion and beauty should be very different for images versus for music, which is why stimulus category is a critical factor in the present study.

### Current study

The present study focuses on the following questions:

1. *How much does the emotion a subject perceives in an object affect beauty judgment?* The literature has worked to establish how and why we might gain pleasure from art objects that evoke various emotions (especially negatively valenced ones), but we are interested in measuring how much object emotion affects beauty.
2. *In what ways does the present mood of a subject affect the beauty judgment they cast on an object?* Are we more capable of appreciating the beauty of a stimulus (and more likely to rate it as beautiful) while in a particular emotional state? Do we have a preference for mood congruence during music listening, as Taruffi and Koelsch (2014) found, or when viewing images?
3. *How differently do positively and negatively valenced emotions affect beauty judgment?* Most of the literature on beauty and emotion focuses only on sadness. Ishizu and Zeki (2017) examine both happy and sad stimuli, without emphasizing differences in magnitude of aesthetic response.
4. *How differently do emotions affect beauty judgments for images versus music?* Only one of the studies reviewed above addresses these differences directly (Miu et al., 2016), and it relies on participants’ recalled experiences with art rather than their immediate reactions to stimuli.
5. *Does the amount of time the subject spends with the object affect the relationship between beauty and emotion?* From a fraction of a second to many seconds, aesthetic judgment is independent of duration (Belfi et al., 2018; Brielmann, Vale, & Pelli, 2017), but perhaps the interactions between beauty and emotion do depend on time.

This study presents participants with a mood questionnaire followed by a set of songs and images, asking them to rate each in terms of their perceived happiness, sadness, and beauty. It includes a mood intervention to broaden the range of participant moods and to help identify the potential causal (not just correlational) relationship between subject emotion and object beauty. Although most of the literature on emotion and aesthetic response attempts a general explanation for why people enjoy stimuli like sad music, these debates also require answers to “What?” and “How much?” The present study aims to provide these answers, contributing empirical data that help characterize the relationship between emotion and beauty more broadly.

## Methods

### Participants

We recruited 81 participants through Prolific Academic (https://prolific.com/) who took part in the experiment; 69 were included in the analysis, with data from 12 excluded because they failed the attention checks described in the Procedure section. Of the 69, 28 identified as female, 35 as male, 5 as nonbinary, and 1 did not disclose their gender. Forty-one participants identified as White, 8 as Black, 7 as Hispanic, 6 as Asian, 5 as mixed race, and 2 as an other race/ethnicity. Their ages ranged from 18 to 28 years (*M* = 24.0, *SD* = 2.7). We limited the participant age range because we needed the stimulus set to be relevant to as much as the group as possible (i.e., show variation in beauty ratings, including high ratings), and music and visual art preferences vary widely across age groups. All participants spoke English as their first language, were U.S. nationals, had no hearing impairments, and had normal or corrected-to-normal vision. In terms of highest level of education, 21 participants had completed high school at the time of the study, 20 had completed some college, 10 had an associate's degree, 15 had a bachelor's degree, and 3 had a master's degree. On average, participants had 2.9 ± 5.2 years of visual art training and 3.2 ± 4.9 years of music training. Fifteen participants had 4 or more years of visual art training and 22 had 4 or more years of music training. All participants gave informed consent in accordance with the declaration of Helsinki. This experiment was approved by the New York University Committee on Activities Involving Human Subjects (UCAIHS; IRB-FY2020-4100).

### Stimuli

We assembled a stimulus set containing 12 visual art images, 12 nature photographs, and 24 song excerpts, taken from a variety of Internet sources and selected to satisfy several criteria. First, we aimed to include stimuli that would be judged as very beautiful so that as many participants as possible would experience intense beauty in response to the stimuli. Second, for each stimulus type (art images, nature photos, and music), we aimed to include a balanced proportion of stimuli that participants would consider very happy, very sad, or neither. Third, we wanted all stimuli to be unfamiliar to most participants to avoid familiarity introducing a confound. Fourth, because the survey involves two blocks of 24 trials each, one before and one after a mood induction phase, we wanted the sets of stimuli shown in each block to be as similar as possible in terms of styles and perceived emotions represented. This strategy would help to ensure there were no consequential differences in these main measures before and after mood induction caused by stimulus assignment.

To achieve this goal, we first compiled a set of several hundred images and songs that we then piloted by asking a separate group of 15 participants to rate them in terms of perceived beauty, liking, happiness, sadness, and familiarity. These participants were recruited the same way as those who took the main survey (see the Participants section). We compiled this initial large set of stimuli in the following way. To capture visual art that would appeal to a broad range of people, we selected artworks in various popular traditional styles, including Dutch florals and Impressionist and Romantic landscapes. We took nature photos with a broad range of subjects and color palettes from the open-source images site Unsplash (https://unsplash.com/). To select music stimuli that had the potential to elicit an intense beauty response in most or all participants, we first referenced the Billboard Top 100 artist list (https://www.billboard.com/charts/artist-100/), and we then searched Dubolt (https://dubolt.com/) for lesser known artists with similar sounds to those on the Billboard Top 100. Dubolt is a website partnered with Spotify that generates playlists based on artists or songs. Users can tailor playlists according to a variety of music features, like popularity, energy, tempo, and mood. These features enabled us to search for songs fitting our criteria. We selected songs from this list that we expected would be the least familiar of these popular options (songs with lower play counts on Spotify, like those released by emerging artists that were recommended on popular artist pages) to avoid a familiarity bias. Neither the image nor the song sets were selected to be representative of all genres and geographies. We limited visual artwork to predominantly Western representational works, and we limited music to pop (defined broadly), all in an attempt to eliminate geography and genre as confounds. We did not want within-participant beauty rating variance to come from genre; we wanted it to come from the emotion factors of interest to us.

Once songs were selected, we downloaded and trimmed them to 20 seconds each, selecting excerpts we felt were emotionally evocative (often a musical hook or a portion of the chorus) and that had a drop in loudness at the start and end of the clip to avoid abrupt starts and stops that might reduce beauty and liking ratings unnecessarily. We then trimmed each 20-second song clip down to 2 seconds to create an additional set of 24 clips, again taking from the 20-second clips 2-second clips that were especially emotionally evocative.

During presentation of the image stimuli, the screen background was white, the images were horizontally centered, and the images were sized to have a height of 600 pixels (with varying width depending on aspect ratio) for consistency across the set.

To satisfy our fourth criterion (that stimuli shown before and after the mood induction for each participant would be as similar as possible in terms of styles and perceived emotions represented), all 48 stimuli were selected in pairs that were similar in style and subject and that expressed similar emotions according to pilot data. For images, pairs were also in the same orientation (vertical vs. horizontal). Each participant saw one stimulus from each pair in block 1 and the other in block 2 so that the range of style–emotion combinations was the same across both blocks for every participant. Stimuli within each pair were pseudorandomly assigned to block 1 or block 2 in the following way: participants were randomly assigned one of six versions of the survey, where each survey had different combinations of stimuli in each block, but no one block contained both stimuli from a given pair (otherwise the six surveys were identical).

**Figure 1.**
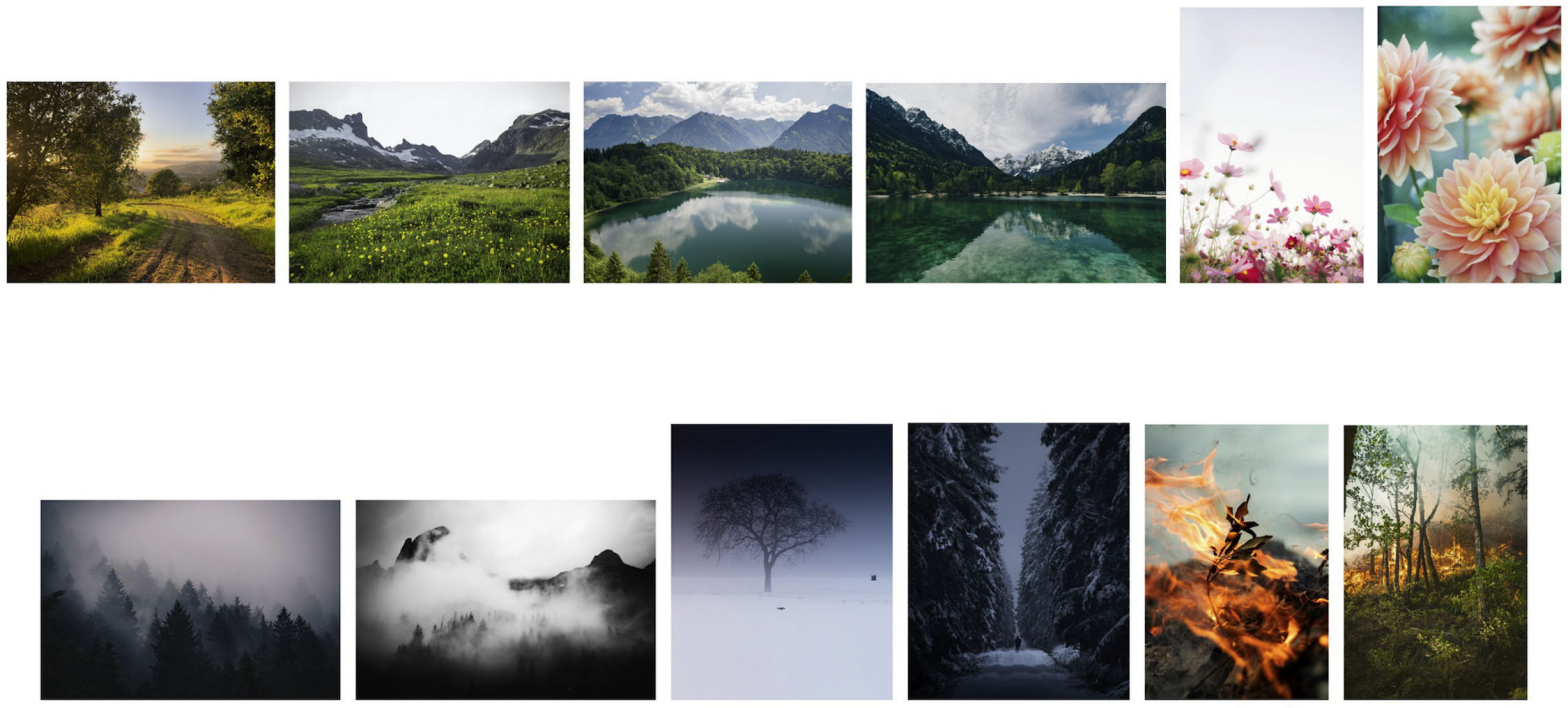
All 12 nature photographs taken from Unsplash (https://unsplash.com/), sorted approximately by mean happiness rating (from pilot data) in descending order. The order is adjusted so that image pairs are side by side.

After collecting pilot data from 15 participants on this large set of stimulus candidates, we selected our final 12 visual art images, 12 nature photographs, and 24 song excerpts (each with 20-second and 2-second versions) to fit the criteria outlined above. All stimuli and summary statistics of their ratings can be found here: https://osf.io/e8uaq/. Nature photographs are shown in Figure 1.

### “Brief” and “prolonged” stimulus durations

To test the effect of duration, stimulus presentation was either brief or prolonged. Each stimulus was presented on its own page before the page of questions about it (see Procedure). After 2 seconds (brief) or 20 seconds (prolonged), the stimulus page automatically advanced to the question page. Participants were given the following instructions before each stimulus trial block (note that the bracketed words were never shown; they indicate that the instructions refer to either images or songs and 2- or 20-second durations):

“In this section of the survey, you will be asked questions about 12 images [song clips]. You will view each image [song clip] on its own page for 2 [20] seconds, and then you will advance automatically to a page with questions about that image [song].

“We encourage you to immerse yourself in each image [song]. As you view [hear] them, consider the following:

• What about the image [song] draws you in?
• Do you enjoy the image [song]?
• Does the image [song] move you?”

Each of the 81 participants (before exclusions) took one of four surveys, and each survey contained two stimulus types: either brief or prolonged images and either brief or prolonged songs. Table 1 shows the number of participants who completed each version of the survey.

**Table 1.** Count of participants presented with each stimulus type.

		Images	
		Brief (2 s)	Prolonged (20 s)
Songs	Brief (2 s)	*N* = 20	*N* = 20
	Prolonged (20 s)	*N* = 20	*N* = 21

### Mood induction videos

We presented a mood induction video halfway through the survey to increase participants’ happiness or sadness level or to leave their mood unchanged (control). A primary goal of this study is to understand the possible causal (not just correlational) relationship between subject emotion and beauty. To speak to cause, we did an intervention. This intervention ensured that the majority of participants rated stimuli while in two different emotional states, meaning that the effects of subject emotion we report are not merely effects of individual difference in disposition or another confound, but are indeed effects of subject emotion. The intervention also broadened the range of participant moods.

For the happiness and sadness induction, we piloted numerous videos other studies have used for this purpose (e.g., Fernández-Abascal & Martín Díaz, 2013; Fernández-Aguilar, Ricarte, Ros, & Latorre, 2018; Martínez-Rodrigo et al., 2019; Schaefer, Nils, Sanchez, & Philippot, 2010; Starcke, Mayr, & von Georgi, 2021). We found that videos we sourced ourselves from YouTube were more successful in inducing happy and sad moods, likely because the successful videos were chosen specifically for the 18- to 28-year-old participant demographics most prevalent in Prolific. We piloted more than 60 videos on 461 total participants, recruited in the same way we recruited participants for the main survey, as described in the Participants section. The mood induction video pilot survey consisted of the PANAS questionnaire (Watson, Clark, & Tellegen, 1988), followed by a mood induction video, followed by the PANAS questionnaire again. The pilot survey can be found here: https://osf.io/e8uaq/. Each of the 461 participants saw only one of the approximately 60 videos tested, approximately 8 participants per video. (We could more easily test a large sample for this pilot than the main study because it lasted 4 to 7 minutes rather than 35 to 50 minutes for the main study.) After analyzing changes in PANAS questionnaire measures before and after mood induction during the pilot survey, we selected the following videos for mood induction in the main study:

• Happiness induction: Mixtape Medley with Ariana Grande and Kelly Clarkson (https://www.youtube.com/watch?v=EJye4M1JMYI).
• Sadness induction: Jack's death scene from *Titanic* (1997) (https://www.youtube.com/watch?v=w6OzanamcI8).
• Neutral mood induction (control): Clip from the film *Blue* (1993) during which the character Olivier shifts papers on a desk, sourced from the film clip database put together by Schaefer et al. (2010).

In the main study, participants were randomly assigned either the happy, sad, or neutral mood induction video. Twenty-seven participants were shown the happy mood induction video, 26 were shown the sad mood induction video, and 28 were shown the neutral mood induction video.

### Measures

Stimulus trial questions are listed below.

1. How much do you like this image [song]? (*7-point scale from “not at all” to “a lot”*).
2. How much beauty do you feel from this image [song] right now? (*7-point scale from “none at all” to “a lot”*).
3. How much happiness does this image [song] evoke? (*7-point scale from “none at all” to “a lot”*).
4. How much sadness does this image [song] evoke? (*7-point scale from “none at all” to “a lot”*).
5. How familiar are you with this particular image [song]? (*7-point scale from “not at all” to “very familiar”*).
6. How familiar are you with images [songs] like this one? (*7-point scale from “not at all” to “very familiar”*).

We used the Positive and Negative Affect Score (PANAS) mood questionnaire (Watson et al., 1988) to collect data about participants’ present mood. To the PANAS questionnaire we appended two additional emotion items—happy and sad—so that we could leverage both the PANAS positive and negative affect scores, as well as simple measures for happiness and sadness, because the questions we asked about the emotion evoked by stimuli asked about happiness and sadness. The questionnaire asked participants to “Indicate to what extent you feel this way right now, that is, at the present moment,” and all emotion items used a 5-point Likert scale from “very slightly or not at all” to “extremely”.

We also collected the following demographic measures from each participant: age, gender, highest level of education, race/ethnicity, visual art and music training questions, the AReA (Schlotz et al., 2021), the BMRQ (Mas-Herrero et al., 2013), and the Questionnaire of Cognitive and Affective Empathy (QCAE) (Reniers et al., 2011).

### Survey

All surveys (including pilot and main surveys) were programmed on Qualtrics (https://www.qualtrics.com/). Participants were instructed to use a desktop computer or laptop to complete the study, not a smartphone or tablet, but we did not verify compliance.

### Procedure

**Figure 2.**
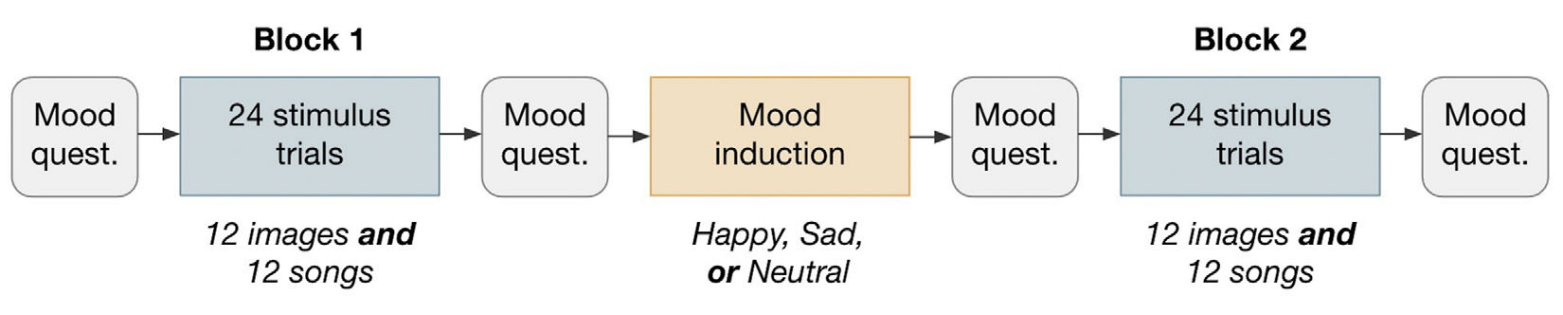
Survey structure. Participants were randomly assigned either a happy, sad, or neutral mood induction video. In Blocks 1 and 2 of stimulus trials, the order of presentation of each stimulus type was randomized (either all images or all songs first). The mood questionnaire used was the PANAS (Watson et al., 1988).

After giving consent and reading brief instructions about the contents, participants rated the 24 songs, 12 art images, and 12 nature photographs and took the PANAS mood questionnaire following the structure shown in Figure 2. Participants rated all the image stimuli or all the song stimuli first within each block with the order of stimulus type presentation counterbalanced and the presentation order of each individual stimulus randomized.

We included two attention checks. One was in the form of an additional auditory stimulus trial, which included a voice recording in place of a song clip with the same stimulus trial questions listed for all other auditory stimuli. The recording instructed participants to mark the rightmost answer choice for every question on the page. This attention check also ensured participants’ audio was functional. Additionally, at the end of the survey, we asked participants how attentive they had been throughout the survey on a 7-point scale from “not at all attentive” to “very attentive”. The complete survey can be found here: https://osf.io/e8uaq/.

### Analysis

All analyses were conducted using R (Version 4.2.0) in RStudio. We excluded all participants who failed the audio attention check. First, we computed positive and negative affect scores for each PANAS mood questionnaire for each participant (Watson et al., 1988). Each participant's ratings for each stimulus was associated with responses from the mood questionnaire they took before seeing that block of stimuli. Next, we tested whether the mood induction was successful. We performed two-tailed paired *t*-tests on the following measures from the mood questionnaires participants completed before and after viewing the mood induction video: positive affect score, negative affect score, happiness rating, and sadness rating.

Next, we fit linear mixed-effects models and calculated likelihood ratios using the *lme4* (Bates, Maechler, Bolker, & Walker, 2015) and *lmerTest* (Kuznetsova, Brockhoff, & Christensen, 2017) packages in R. We used beauty ratings as the dependent variable for each of the four stimulus types: prolonged (20-second) and brief (2-second) images and songs. All linear mixed-effects models included fixed factors for object happiness, object sadness, subject happiness, and subject sadness. We also looked at the effect of the following four interactions on beauty ratings: Object happiness × Subject happiness, Object happiness × Subject sadness, Object sadness × Subject happiness, and Object sadness × Subject sadness, where object emotion is provided by the appropriate Likert rating after each presentation of a particular stimulus and subject emotion is provided by the happiness/sadness simple rating provided by each participant in the mood questionnaire before each block. In this way, we factor the mood induction into the analysis using the participant's self-reported mood ratings, rather than the mere fact that we showed them a video intended to induce mood. This measurement method avoids the presumption that the mood induction had the same effect for each individual. We included random intercepts in the linear mixed-effects models for both stimulus and participant, which account for natural random variation in ratings across many stimuli and participants. We also tested linear mixed-effects models using positive and negative affect scores obtained from the PANAS questionnaire in place of happiness and sadness ratings (see Supplementary Materials). We used the *r.squaredGLMM()* function (Johnson, 2014) from the MuMIn package (version 1.40.4) in R to compute conditional coefficients of determination for all of our linear mixed-effect models. This coefficient represents the percent of data variation the model explains and is reported in the caption of each linear mixed-effects model result table in our manuscript and Supplementary Materials. Using a linear model as proxy, we calculated the sensitivity of our sample size for each linear model, which indicates a 95% chance of detecting effect sizes as small as 0.02. The data and code can be found here: https://osf.io/e8uaq/.

## Results

We excluded results from the 12 participants who failed the audio attention check. The minimum attentiveness rating given was 4 on a 7-point scale from not at all to very attentive (given by just one participant), and we chose not to exclude results from this participant. Across all 81 participants, attentiveness was self-rated at 6.4 ± 0.8 (*M* ± *SD*).

Results from the remaining 69 participants are described, split by stimulus type. The linear mixed-effects models use happiness and sadness ratings from the mood questionnaires rather than the PANAS positive and negative affect scores so that object and subject emotion measures are analogous. Model performance metrics are similar for the subject happiness/sadness models and PANAS positive/negative affect models (see Supplementary Materials). Additionally, the linear mixed-effects models we show here measure the effect of four object × subject emotion interactions (Object happiness × Subject happiness, Object sadness × Subject sadness, Object happiness × Subject sadness, and Subject happiness × Object sadness) on beauty, but models pared down to include only significant effects can be found in the Supplementary Materials.

### Prolonged (20-second) songs

**Figure 3.**
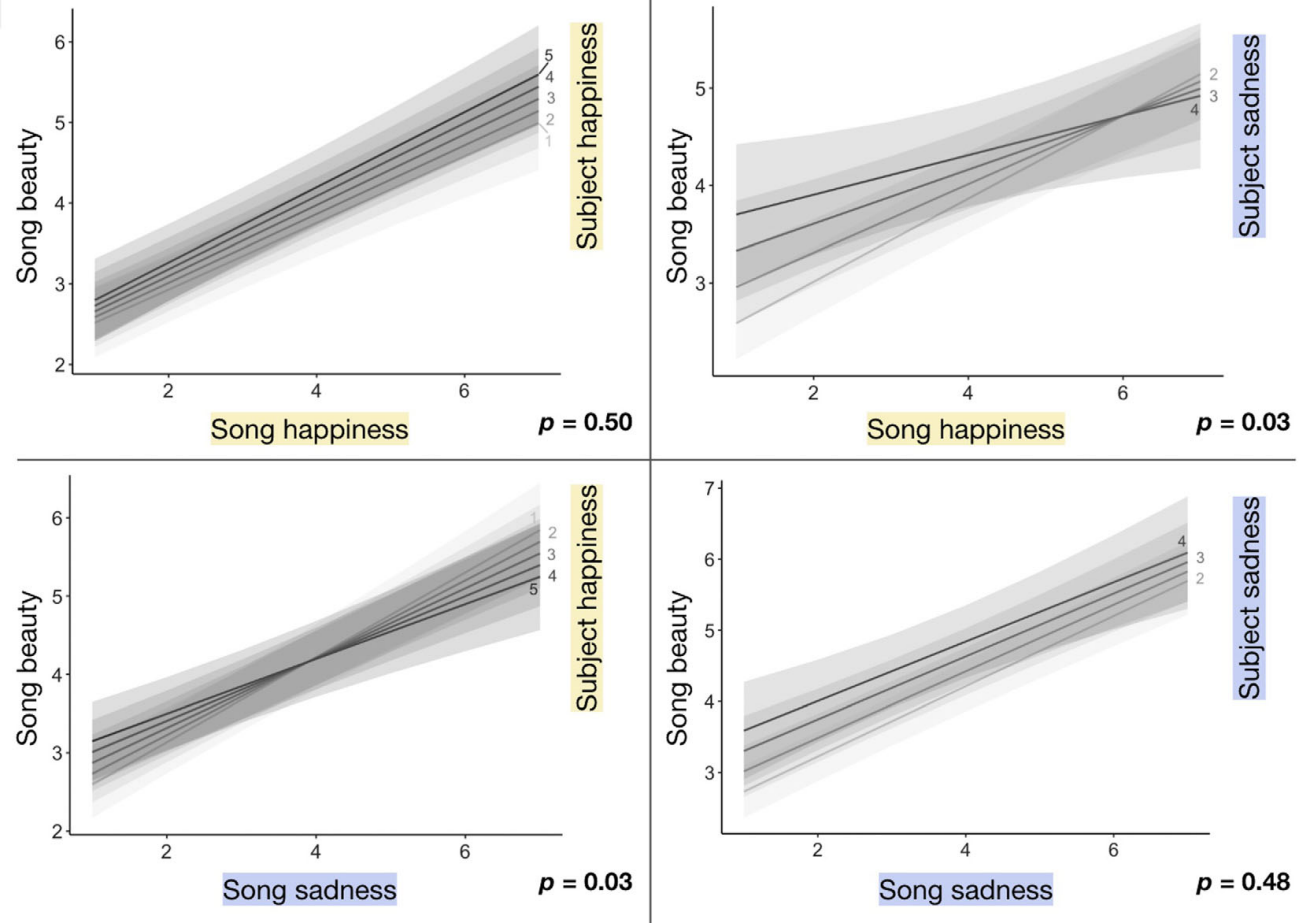
Plots show the effects of each Object × Subject emotion interaction on prolonged (20-second) song beauty. Regression lines are split by the happiness or sadness rating (5-point Likert scale from not at all to very happy or sad) the participant gave themself before the stimulus trial (i.e., the subject's emotion). *p*-values correspond with the Object × Subject emotion interaction.

The linear mixed-effects model for prolonged songs (Table 2) shows that both the happiness and sadness ratings participants gave songs predict increases in beauty ratings, and when the participant is sadder or happier, respectively, these effects are diminished slightly. All else being equal, one additional object happiness point results, on average, in a 0.47-point beauty increase, and one additional object sadness point results, on average, in a 0.61-point beauty increase. Subject emotion also affects beauty. All else being equal, one additional subject sadness point results, on average, in a 0.50-point beauty increase. Incongruent object and subject emotions diminish beauty, with a point of subject sadness diminishing the positive effect of object happiness on beauty by 0.07, on average, and with a point of subject happiness diminishing the positive effect of object sadness on beauty by 0.05, on average. The four plots in Figure 3 display the four interactions of interest, with song emotion on the *x*-axis, beauty on the *y*-axis, and trendlines split by subject emotion. Trendlines are positively sloped and visualize the difference in slopes associated with different subject emotion ratings. These results quantify the effects of song happiness and sadness on song beauty (questions 1 and 3 listed in the Introduction). They show a direct effect of subject emotion on song beauty and an interaction between song emotion and subject emotion on song beauty (question 2).

**Table 2.** Linear mixed-effects model for prolonged (20-second ) songs (*N* = 35). The model explains 64% of the variance in the data. Bold values indicate statistical significance.

Random effects	Variance	*SD*			
Participant	0.599	0.774			
Stimulus	0.175	0.419			
Fixed effects	Estimate	*SE*	*df*	*t*	*p*-value

(Intercept)	0.37	0.50	585	0.74	0.462
**Object happiness**	**0.47**	**0.09**	**821**	**5.30**	**<** **0.001**
Subject happiness	0.15	0.11	768	1.39	0.164
**Object sadness**	**0.61**	**0.09**	**824**	**6.55**	**<** **0.001**
**Subject sadness**	**0.50**	**0.21**	**797**	**2.34**	**0.019**
Object happiness:subject happiness	0.01	0.02	821	0.68	0.498
Object sadness:subject sadness	−0.03	0.04	809	−0.71	0.481
**Object happiness:subject sadness**	**−0.07**	**0.03**	**820**	**−2.15**	**0.032**
**Object sadness:subject happiness**	**−0.05**	**0.02**	**818**	**−2.21**	**0.027**

### Brief (2-second) songs

**Figure 4.**
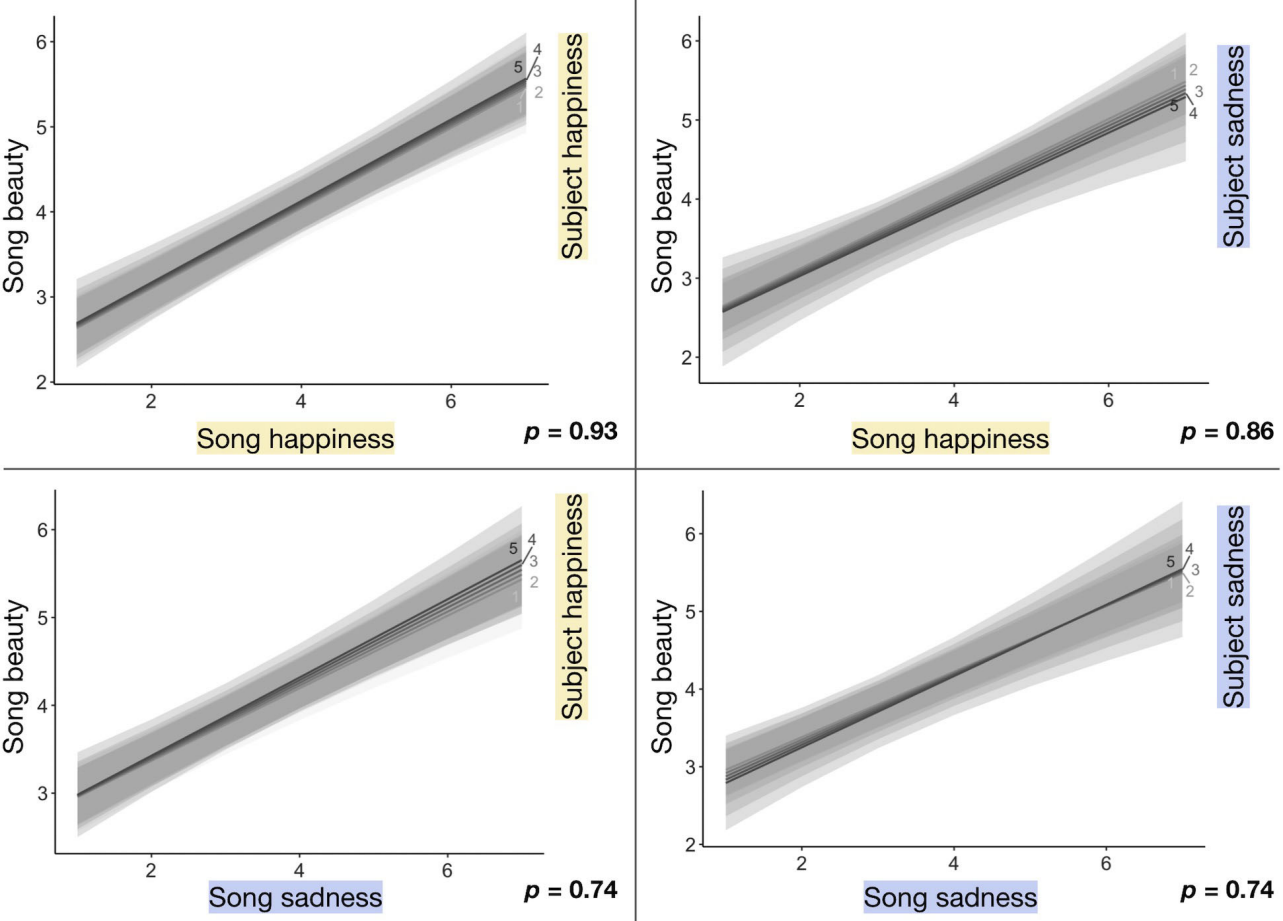
Plots show the effects of each Object × Subject emotion interaction on brief (2-second) song beauty. Regression lines are split by the happiness or sadness rating (5-point Likert scale from not at all to very happy or sad) the participant gave themself before the stimulus trial. *p*-values correspond with the Object × Subject emotion interaction.

The linear mixed-effects model for brief songs (Table 3) also shows a significant positive effect of perceived object emotion (both for object happiness and sadness ratings) on beauty but—very differently from prolonged songs—no effect of subject emotion on beauty. One additional object happiness point results in an average 0.47-point beauty increase, and one additional object sadness point results in an average 0.40-point beauty increase. Although incongruent object and subject emotions diminish the beauty of prolonged songs, for brief songs there were no significant interactions between subject and object emotion. This difference is visualized in Figure 4, where, unlike in Figure 3, trendline slopes are nearly identical across subject emotion ratings. These results directly address question 5 in the Introduction—namely, that duration does affect the relationship between emotion and beauty of songs.

**Table 3.** Linear mixed-effects model for brief (2-second) songs (*N* = 34). The model explains 61% of the variance in the data. Bold values indicate statistical significance.

Random effects	Variance	SD			
Participant	0.451	0.671			
Stimulus	0.110	0.331			
Fixed effects	Estimate	*SE*	*df*	*t*	*p*-value

**(Intercept)**	**1.17**	**0.48**	**564**	**2.45**	**0.015**
**Object happiness**	**0.47**	**0.09**	**800**	**5.38**	**<** **0.001**
Subject happiness	−0.01	0.14	777	−0.05	0.958
**Object sadness**	**0.40**	**0.09**	**797**	**4.34**	**<** **0.001**
Subject sadness	−0.04	0.16	694	−0.22	0.823
Object happiness:subject happiness	0.00	0.02	796	0.09	0.932
Object sadness:subject sadness	0.01	0.03	794	0.33	0.743
Object happiness:subject sadness	−0.01	0.03	786	−0.17	0.862
Object sadness:subject happiness	0.01	0.02	796	0.33	0.740

### Prolonged (20-second) images

**Figure 5.**
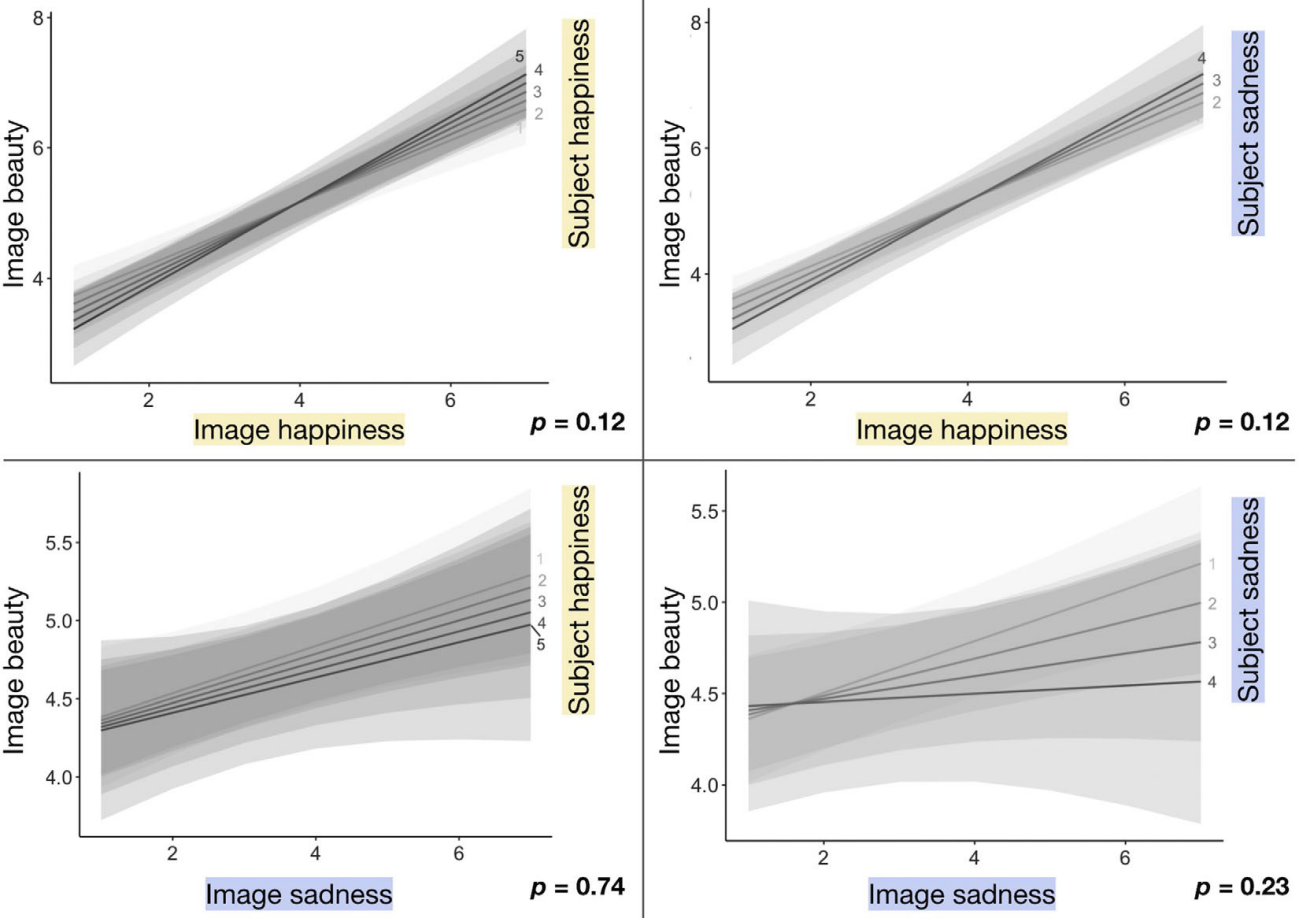
Plots show the effects of each Object × Subject emotion interaction on prolonged (20-second) image beauty. Regression lines are split by the happiness or sadness rating (5-point Likert scale from not at all to very happy or sad) the participant gave themself before the stimulus trial. *p*-values correspond with the Object × Subject emotion interaction.

The linear mixed-effects model for prolonged images (Table 4) shows a significant effect of perceived object emotion (both object happiness and sadness ratings) on beauty but no effect of subject emotion on beauty. One additional object happiness point results in an average 0.38-point beauty increase, and one additional object sadness point results in an average 0.20-point beauty increase. There were no significant interactions between subject and object emotion. So, rather than all four of the emotion factors examined (object happiness and sadness and subject happiness and sadness) having an impact on beauty, as we saw for prolonged songs, the only factor strongly affecting prolonged image beauty is object happiness. The top two plots in Figure 5 resemble those in Figure 4 (brief songs), but the bottom two differ starkly, with nearly flat trendlines associated with the relationship between image sadness and beauty. Here we see a substantive difference in results for images versus songs, answering question 4 in the Introduction and giving further insight into questions 1 through 3.

**Table 4.** Linear mixed-effects model for prolonged (20-second) images (*N* = 33). The model explains 54% of the variance in the data. Bold values indicate statistical significance.

Random effects	Variance	SD			
Participant	0.295	0.543			
Stimulus	0.188	0.433			
Fixed effects	Estimate	*SE*	*df*	*t*	*p*-value

**(Intercept)**	**3.04**	**0.64**	**538**	**4.79**	**<** **0.001**
**Object happiness**	**0.38**	**0.10**	**754**	**3.75**	**<** **0.001**
Subject happiness	−0.14	0.18	595	−0.80	0.425
**Object sadness**	**0.20**	**0.10**	**748**	**1.99**	**0.047**
Subject sadness	−0.09	0.20	536	−0.47	0.642
Object happiness:subject happiness	0.04	0.03	757	1.57	0.117
Object sadness:subject sadness	−0.04	0.03	761	−1.20	0.229
Object happiness:subject sadness	0.05	0.03	753	1.56	0.119
Object sadness:subject happiness	−0.01	0.03	736	−0.33	0.739

### Brief (2-second) images

**Figure 6.**
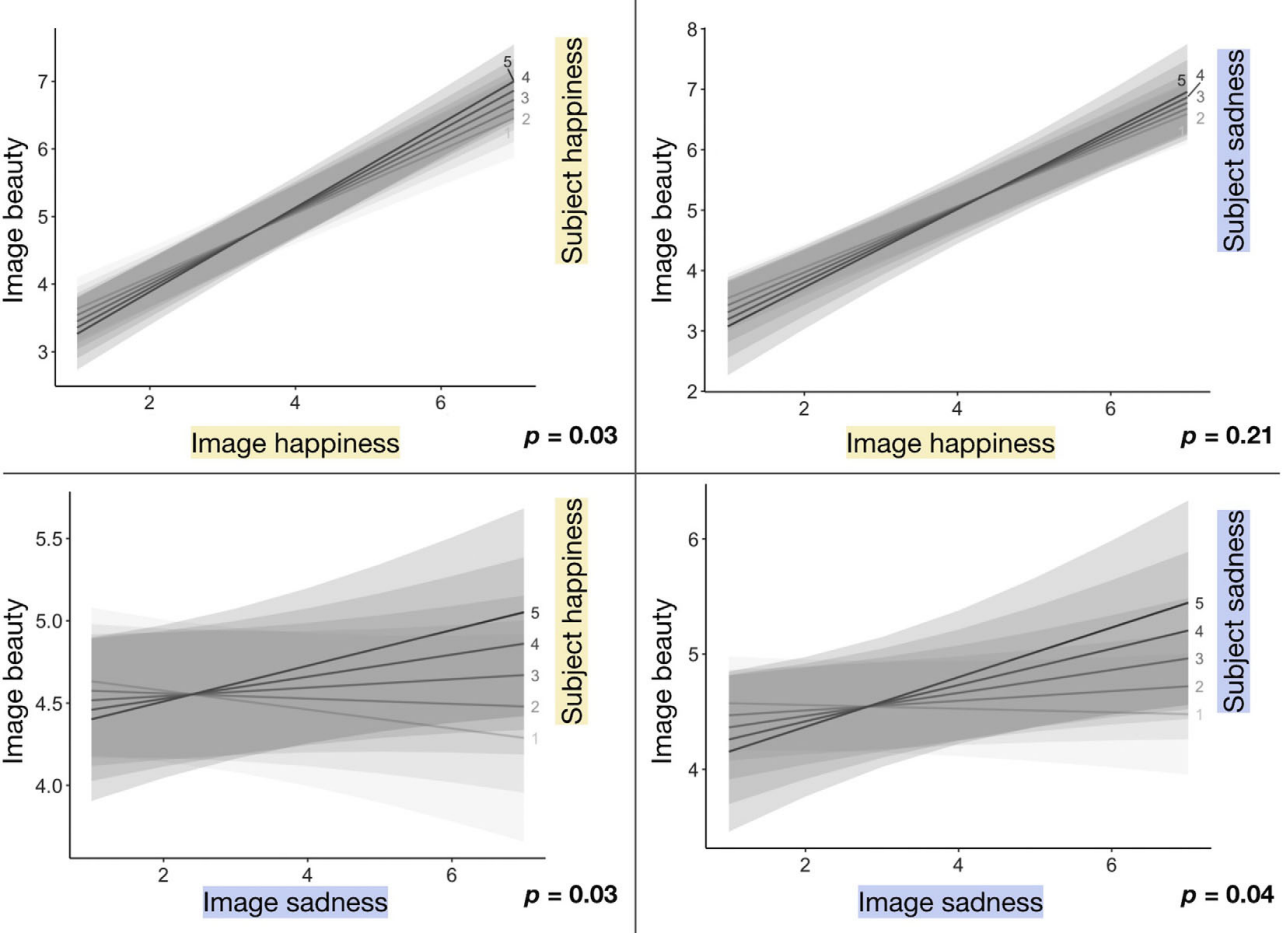
Plots show the effects of each Object × Subject emotion interaction on brief (2-second) image beauty. Regression lines are split by the happiness or sadness rating (5-point Likert scale from not at all to very happy or sad) the participant gave themself before the stimulus trial. *p*-values correspond with the Object × Subject emotion interaction.

The linear mixed-effects model for brief images (Table 5) shows that the happiness ratings participants gave images again had a significant effect on beauty ratings. All else being equal, one additional object happiness point results in a 0.40-point beauty increase, on average. Unlike for prolonged images, object sadness does not have a significant effect on brief image beauty. One point of subject happiness, however, now predicts a 0.21-point reduction in beauty, on average. There are also significant positive interactions between image sadness and subject happiness and sadness, but neither object sadness nor subject sadness affect brief image beauty on their own. Although perceived object sadness boosts prolonged image beauty regardless of subject emotion, object sadness boosts brief image beauty only when the subject is emotional. Figure 6 visualizes this—image happiness predicts an increase in beauty regardless of subject emotion, but image sadness only predicts an increase in beauty when the subject is emotional. This verifies the modality-level difference in the effect of emotion on beauty (question 4 in the Introduction), and it shows subtle duration-level differences in the effect of subject emotion on image beauty (question 5).

**Table 5.** Linear mixed-effects model for brief (2-second) images (*N* = 36). The model explains 67% of the variance in the data. Bold values indicate statistical significance.

Random effects	Variance	SD			
Participant	0.770	0.877			
Stimulus	0.319	0.564			
Fixed effects	Estimate	*SE*	*df*	*t*	*p*-value

**(Intercept)**	**3.76**	**0.45**	**504**	**8.40**	**<0.001**
**Object happiness**	**0.40**	**0.08**	**851**	**4.98**	**<0.001**
**Subject happiness**	**−0.21**	**0.10**	**836**	**−2.07**	**0.039**
Object sadness	−0.16	0.08	851	−1.89	0.059
Subject sadness	−0.27	0.17	846	−1.62	0.107
**Object happiness:subject happiness**	**0.04**	**0.02**	**835**	**2.17**	**0.030**
**Object sadness:subject sadness**	**0.06**	**0.03**	**835**	**2.10**	**0.036**
Object happiness:subject sadness	0.03	0.03	836	1.26	0.209
**Object sadness:subject happiness**	**0.04**	**0.02**	**830**	**2.17**	**0.030**

### Effect of stimulus duration

We ran a Pearson correlation analysis and found strong positive correlations between the mean beauty (*r* = 0.95), happiness (*r* = 0.97), and sadness (*r* = 0.97) ratings of 20-second versus 2-second versions of the same images and between the mean beauty (*r* = 0.82), happiness (*r* = 0.95), and sadness (*r* = 0.95) ratings of 20-second versus 2-second versions of the same songs (*p* < 0.001). We also ran a two-tailed two-sample *t*-test on these mean stimulus ratings, finding significant but small differences in emotion ratings just for image sadness and song happiness. Prolonged image sadness was rated 0.41 points higher on average than brief image sadness (*p* < 0.001), and prolonged song happiness was rated 0.22 points lower on average than brief song happiness (*p* = 0.01). These results are shown in Tables 6 and 7.

**Table 6.** Comparison of mean beauty, happiness, and sadness ratings for brief (2-second) versus prolonged (20-second) images. The left columns show the results of a two-tailed two-sample *t*-test (positive differences indicate higher brief image ratings), and the rightmost two columns show the results of a Pearson's correlation analysis.

Stimulus trial item	Mean difference	*t*	*p*-value	*df*	*r*	*p*-value
Beauty	−0.003	−0.04	0.97	1653	0.94	<0.001
Happiness	0.0007	0.008	0.99	1653	0.97	<0.001
Sadness	0.41	4.38	<0.001	1653	0.97	<0.001

**Table 7.** Comparison of mean beauty, happiness, and sadness ratings for brief (2-second) versus prolonged (20-second) songs. The left columns show the results of a two-tailed two-sample *t*-test (positive differences indicate higher prolonged ratings), and the rightmost two columns show the results of a Pearson's correlation analysis.

Stimulus trial item	Mean difference	*t*	*p*-value	*df*	*r*	*p*-value
Beauty	0.16	1.80	0.07	1654	0.82	<0.001
Happiness	−0.22	−2.50	0.01	1654	0.95	<0.001
Sadness	−0.01	−0.15	0.88	1654	0.95	<0.001

### Effect of art and music expertise and reward sensitivity

Participants reported their visual art and music training (number or years and type) and their sensitivity to aesthetic reward, as assessed by the AReA and BMRQ (Mas-Herrero et al., 2013; Schlotz et al., 2021). We added the visual art version of these metrics (number of years of art training and AReA score) to our linear mixed-effects models for images, and we added the music version of these metrics (number of years of music training and BMRQ score) to our linear mixed-effects models for songs. Results can be found in the Supplementary Materials. These factors only show significant effects on the beauty of brief images and songs. For brief songs, 1 point of change in BMRQ corresponds to 0.02 additional beauty points (*p* = 0.03), on average. For brief images, 1 point of change in AReA corresponds with 0.04 additional beauty points (*p* = 0.02), on average. Unlike findings from Van Paasschen, Bacci, and Melcher (2015) and others, we see no effect of visual art and music training on beauty judgments. The linear mixed-effects models that include AReA or BMRQ and visual art or music training explain just 0 to 1% more variance than the models without them do.

### Effect of cognitive and affective empathy

Participants took the QCAE (Reniers et al., 2011) near the end of the survey. To compare the effects of emotion on beauty (both direct effects and interactions) directly for relatively high versus relatively low empathy groups of participants, we split the dataset into two such groups: low empathy, where participants scored below the total group mean QCAE score, and high empathy, where participants scored above it. For prolonged songs, the low empathy linear mixed-effects model (*N* = 15) shows no effect of subject emotion on prolonged song beauty at all. In contrast, for the high empathy dataset (*N* = 20), one additional subject happiness point results in an average 0.56-point beauty increase and one additional subject sadness point results in an average 0.67-point beauty increase. Additionally, for brief images, the low empathy data (*N* = 15) show a negative effect of subject sadness on beauty (a 0.56-point reduction in beauty), whereas the high empathy data (*N* = 19) show no effect of subject sadness. We did also add QCAE score as a fixed effect to the original models to examine its direct relationship to beauty. For brief images and songs, 1 point of change in QCAE score corresponded to 0.01 (*p* = 0.02) and 0.02 (*p* = 0.001) additional beauty points, on average, respectively. There was no effect for prolonged images and songs. All of these results can be found in the Supplementary Materials.

### Examination of emotion rating space

In addition to verifying that participants perceived a range of happiness and sadness levels in the stimuli (see Supplementary Materials), we were interested in relationships between happiness and sadness ratings themselves and in the relationship between subject emotion and object emotion ratings. First, we analyzed the correlations between happiness and sadness of images, songs, and subjects, finding a Pearson correlation coefficient of −0.27 for subject happiness versus subject sadness (*p* < 0.001), −0.32 for image happiness versus image sadness (*p* < 0.001), and −0.15 for song happiness versus song sadness (*p* < 0.001). Scatterplots that show these relationships as well as plots showing simple beauty, happiness, and sadness rating distributions can be found in the Supplementary Materials. Second, we assessed whether subject emotion affects perceived object emotion, and it does not. We ran two linear mixed-effects models with subject happiness and subject sadness as fixed effects, participant and stimulus as random intercepts, and either object happiness or object sadness as the dependent variable. All *p*-values for both models were above 0.05 with *N* = 69.

### Manipulation check

We compared participants’ responses to mood questionnaires before and after viewing mood induction videos to gauge effectiveness of the mood induction during the main study. Tables 8^9^ to 10 contain results from two-tailed paired *t*-tests performed on participants’ ratings to the happy and sad items appended to the PANAS mood questionnaire. Results show that the happiness and sadness induction videos both had the desired effect on participants’ happiness and sadness ratings. After the happiness induction, happy ratings showed a 0.54-point increase (*p* < 0.001) on a 5-point scale, and sad ratings showed a 0.17-point decrease (*p* < 0.001) on a 5-point scale. After the sadness induction, happy ratings showed a 1.18-point decline (*p* < 0.001) on a 5-point scale, and sad ratings showed a 0.95-point increase (*p* < 0.001) on a 5-point scale. After the neutral mood induction we used as a control, happy ratings showed a 0.26-point decline (*p* < 0.001) on a 5-point scale, and sad ratings showed a 0.17-point decrease (*p* < 0.001) on a 5-point scale (likely because the video was boring).

**Table 8.** Happiness induction results (*N* = 27) from two-tailed paired *t*-test. Participants rated the happy and sad items we appended to the PANAS mood questionnaire on a 5-point Likert scale from not at all to very happy or sad.

Mood questionnaire item	Mean difference	*t*	*p*-value	*df*
Happiness	0.54	20.16	<0.001	575
Sadness	−0.17	−6.41	<0.001	575

**Table 9.** Sadness induction results (*N* = 26) from two-tailed paired *t*-test. Participants rated the happy and sad items we appended to the PANAS mood questionnaire on a 5-point Likert scale from not at all to very happy or sad.

Mood questionnaire item	Mean difference	*t*	*p*-value	*df*
Happiness	−1.18	−23.52	<0.001	527
Sadness	0.95	21.45	<0.001	527

**Table 10.** Neutral mood induction results (*N* = 28) from two-tailed paired *t*-test. Participants rated the happy and sad items we appended to the PANAS mood questionnaire on a 5-point Likert scale from not at all to very happy or sad.

Mood questionnaire item	Mean difference	*t*	*p*-value	*df*
Happiness	−0.26	−7.73	<0.001	551
Sadness	−0.17	−10.77	<0.001	551

## Discussion

**Figure 7.**
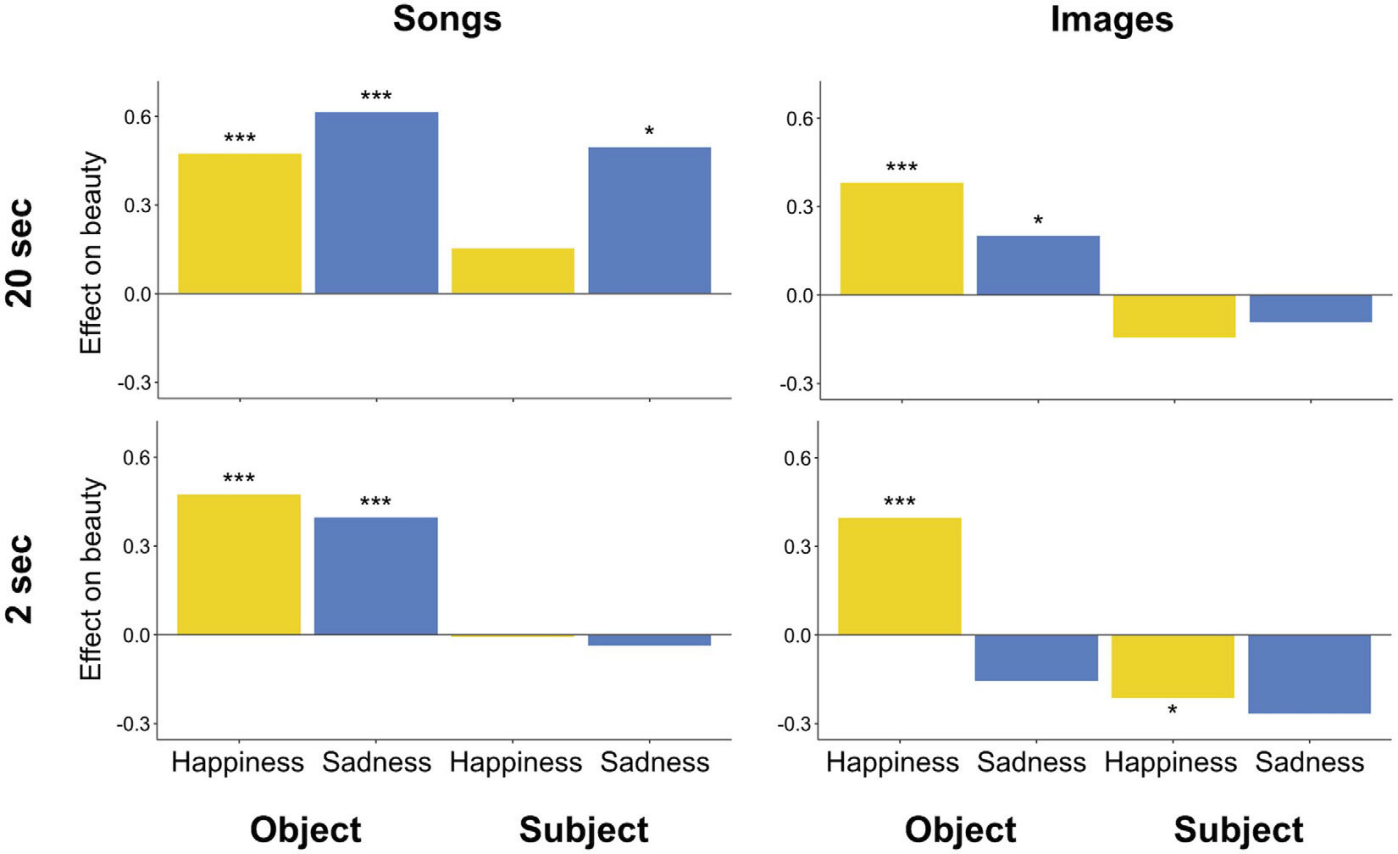
Barplots show beta values from the four linear mixed-effects models, with beauty as dependent variable and object and subject emotions and their interactions as fixed effects (see Results). Models were run separately for each of four stimulus types: 20-second and 2-second songs and images. *p < 0.05; ***p < 0.001.

This study investigates the role of emotion in beauty judgment, accounting for potential differences across four dimensions: locus of emotion (subject and object), emotional valence (happy and sad), sensory modality (visual and auditory), and duration (2 and 20 seconds). In this study, 18- to 28-year-old U.S.-based participants rated 24 images and 24 songs in terms of perceived beauty, liking, perceived happiness, perceived sadness, and familiarity. All participants took the PANAS mood questionnaire several times throughout the survey (see Figure 2). Participants viewed a mood induction video halfway through the survey intended either to make them happier or sadder or to leave their mood unchanged. We find that the interactions between emotion and beauty are different for images and music and are strongly moderated by duration. Major results are illustrated in Figure 7.

### Emotion affects beauty

It is widely assumed that emotion and beauty are intimately related (Hagtvedt et al., 2008; Juslin, 2013; Leder & Nadal, 2014; Menninghaus et al., 2019; Menninghaus et al., 2017; Starr, 2013), and our results provide strong empirical support of this. We see an effect of emotion on beauty across all the dimensions we investigated: both subject and object emotion affect beauty, happiness and sadness affect beauty, emotion affects the beauty of both images and music, and emotion affects beauty differently depending on stimulus duration. Perceived image happiness predicts an increase in image beauty, regardless of duration, and subject emotion has only a minimal effect on image beauty. Perceived song happiness and perceived song sadness predict an increase in song beauty, especially when subject emotion does not oppose perceived object emotion. Subject sadness also boosts song beauty, but all effects of subject emotion on song beauty (including interactions) disappear for short durations.

### Emotion affects the beauty of images and music differently

One aim of this study was to explore differences in the relationship between beauty and emotion across visual and auditory modalities, and we indeed found substantial differences.

For music, both the happiness and sadness listeners perceive in songs predict increased beauty regardless of song duration. For prolonged (20-second) songs, subject sadness predicts increased beauty, and perceived object emotion boosts beauty less when it is incongruent with subject emotion—for example, hearing a sad song while feeling happy. This result affirms previous findings that mood congruence is preferred in music listening (Evans & Schubert, 2008; Taruffi & Koelsch, 2014). One might want to conclude at this point that, counter to philosopher Eduard Hanslick's (1986) claim that the relationship between music and emotion is irrelevant to the beauty of music, perhaps beauty judgment is really about emotion, because object and subject emotion strongly affect the beauty of prolonged songs. Starr (2013) cites suggestions from philosopher and musicologist Peter Kivy (1990), who disagrees with Hanslick about music, emotion, and beauty. Kivy “would resolve all emotional responses to music to the feeling of beauty, so that we are ‘moved by the beauty or perfection of the music’ itself,” suggesting that beauty and feeling are deeply intertwined, so it should not be surprising that basic emotions in object or subject should increase beauty. Yet our results for images and for brief songs temper this view, because subject emotion affects image beauty minimally and does not affect brief song beauty at all, and because perceived image sadness only affects image beauty under certain conditions: if the subject is emotional or if the subject spends more than 2 seconds with the image. Further, the subject emotion effects we see for prolonged songs are only present among participants who score above sample mean on the QCAE (*N* = 20; see Supplementary Materials). Participants with empathy scores below the mean show no effect of subject emotion on prolonged song beauty. This latter result is consistent with studies showing that beauty experiences can involve our faculty of empathy and that individual differences in aesthetic preference can arise from individual differences in empathy (Eerola et al., 2018; Freedberg & Gallese, 2007; Gernot et al., 2018; Wilkinson et al., 2021).

The sadness perceived in the stimulus and the subject's emotional state have smaller effects on the perceived beauty of images than of songs. This result validates the work of the many scholars who have studied the enjoyment of sad music as opposed to sad visual art, yet it also prompts many questions, such as: Why might subject emotion have more influence on the beauty of songs than of images? The study by Miu et al. (2016) might help to address this question. Participants’ most common self-reported reason for engaging with paintings was “self-education” and for music, “mood repair and keeping them company when they felt lonely.” According to their work, engagement with paintings seems to be a more intellectual endeavor, whereas engagement with music seems to be more emotional. These results align with our results, namely, that subject emotion affects beauty minimally for images but strongly for music, and sadder images are only conditionally more beautiful than less sad images, whereas sadder music is much more beautiful than less sad music.

### Duration moderates the effect of subject emotion on beauty

To gauge the effect of stimulus duration on the relationship between emotion and beauty judgment, we compared results for brief (2-second) and prolonged (20-second) stimuli. Visual and auditory stimuli are physically different. Varying music duration requires trimming, while varying image duration does not. Our methods attempt to capture the way duration varies in everyday life.

For prolonged songs, we see a strong effect of subject emotion on beauty: subject sadness predicts an increase in beauty, and incongruent subject and object emotions—for example, feeling happy while hearing a sad song—diminish beauty. However, when songs are trimmed to 2 seconds, the effect of perceived object emotion on beauty remains (object happiness and sadness still boost beauty), but all effects of subject emotion on beauty go away. Stimulus duration also moderates the relationship between the beauty and emotion of images. Happier images are rated as more beautiful regardless of duration. However, sadder brief images are only more beautiful if the subject is emotional, and sadder prolonged images are more beautiful regardless of subject emotion. For both songs and images, then, sadness boosts beauty more for prolonged than short durations.

It is possible that the effect of sadness on beauty might be mediated by another factor: whether or not the subject feels “moved” by the object, as several studies suggest (Hanich, Wagner, Shah, Jacobsen, & Menninghaus, 2014; Menninghaus et al., 2015; Vuoskoski & Eerola, 2017). According to these studies, sadness in the stimulus only amplifies liking if the subject is moved by the object. If we suppose that it takes time to be moved, then this could explain why brief image beauty is not boosted by image sadness but prolonged image beauty is. It is also possible that the way image sadness boosts beauty for prolonged but not brief images is evidence that image sadness is a kind of “difficult beauty.” Bosanquet explains his concept of difficult beauty by saying, “difficult beauty simply gives you too much, at one moment, of what you are perfectly prepared to enjoy if only you could take it all in” (Jacquette, 1984). He would likely consider the images with high sadness ratings to be high in tension, an attribute of difficult beauty that can be appreciated under some circumstances. Yet Bosanquet distinguishes difficult beauty from the difficulty of mathematical problems which require effort over time, where the “paradox about difficult beauty concerns the abilities and inabilities of an individual in the *momentary* [emphasis added] attempt to experience an object as both difficult and beautiful” (Jacquette, 1984). But perhaps the prolonged durations participants spent with sad images did not allow them to solve the images’ difficulty in the way they might solve a math problem; perhaps it gave participants more time to be moved by the images, which in turn would have established a stronger positive relationship between object sadness and beauty (Hanich et al., 2014).

In addition to providing possible empirical insight into Bosanquet's concept of difficult beauty, the effect of stimulus duration might inform models of aesthetic judgment, like those put forward by Leder, Belke, Oeberst, and Augustin (2004), Leder and Nadal (2014), and Pelowski, Markey, Lauring, and Leder (2016). Leder and Nadal (2014) claim that, although “the perceptual aspect of the aesthetic episode takes a fraction of a second, … what makes an experience aesthetic is its long extension in time, which allows for several cycles of feedback and feedforward influence among processes related to perception, cognition and emotion.” Our evidence helps to elucidate one way this might be true: stimulus duration might determine whether and how much particular factors, such as the subject's emotional state, come into play during an aesthetic experience.

Other studies suggest that aesthetic judgment of images and music is independent of duration (Belfi et al., 2018; Brielmann et al., 2017). Our results might seem to contradict this finding; we indeed found that duration affects the relationship between emotion and beauty. However, when we directly assess the relationship between duration and beauty, effects are minimal. Because no participant in our study was given the same stimulus at different durations, we could not replicate their analysis of individuals, but we did analyze the group. Belfi et al. (2018) computed correlations across participants, and we computed correlations across stimuli (see Tables 6 and 7). Our results are qualitatively consistent with those of Brielmann et al. (2017) and Belfi et al. (2018). Brielmann et al. (2017) report that “increasing [image] duration only weakly increased final beauty judgments,” and Belfi et al. (2018) report that “listeners were accurately able to judge how much they liked [a song] excerpt by 750 ms.” Similarly, we did not find a significant difference in beauty ratings for brief versus prolonged images or brief versus prolonged songs, and we only found weak differences in happiness and sadness ratings between durations. To the extent that our studies overlap with those of Brielmann and Belfi, we replicate their findings, but we go on to show that, when subject emotions are measured and when we focus on beauty rather than liking (as Belfi does), large effects of time emerge.

## Conclusions

Emotion affects the beauty of images and music. Both the emotion evoked by the object and the emotional state of the subject affect beauty judgment separately and together. Happier objects are judged more beautiful in all conditions tested, but sadness is more complicated. Sadness boosts beauty more at long durations and more for music than images. For images, object sadness boosts brief image beauty only if the subject is emotional but boosts prolonged image beauty regardless of subject emotion. For songs, object sadness always boosts beauty, and for prolonged songs, subject sadness also boosts beauty. Thus, the interactions between emotion and beauty are different for images and music and are strongly moderated by duration.

## Supplementary Material

Supplement 1
