# Supplementary material for: Emotions of subject and object affect beauty differently for images and music
Source: J Vis. 2023 Nov 16;23(13):6. doi: 10.1167/jov.23.13.6 (PMC10664730; doi:10.1167/jov.23.13.6)
Supplement: Supplement 1 [file jovi-23-13-6_s001.pdf]

Supplementary Materials

Emotions of subject and object affect beauty differently for images and music

Anna Bruns, Maria Pombo, Pablo Ripollés, Denis Pelli

Contents:

1. Linear mixed-effects models that include **only significant effects** (linked here)
2. Linear mixed-effects models that include **liking** in place of beauty (linked here)
3. Linear mixed-effects models that include **familiarity** as a main effect (linked here)
4. Linear mixed-effects models using **PANAS positive and negative affect scores** in place of object happiness and object sadness ratings (linked here)
5. Linear mixed-effects models that include **AReA or BMRQ** and **art or music training** as main effects (linked here)
6. Linear mixed-effects models that include **QCAE** as a main effect (linked here)
7. Linear mixed-effects models that only include data from participants with **above-sample-mean QCAE scores** (linked here)
8. Linear mixed-effects models that only include data from participants with **below-sample-mean QCAE scores** (linked here)
9. Linear mixed-effects models looking at the **effect of prior subject emotion on object emotion ratings** (linked here)
10. Count of participants broken out by stimulus category and mood induction type (linked here)
11. **Correlations between happiness and sadness** of subjects, images, and songs (linked here)
12. Barplots showing **counts of subject happiness and sadness ratings**, split by mood induction group (linked here)
13. Barplots showing **counts of image and song beauty, happiness, and sadness ratings**, split by duration (linked here)

Linear mixed-effects models that include only significant effects

Prolonged (20 sec) songs

Random Effects	*Variance*	*SD*			
Participant	0.61	0.78			
Stimulus	0.18	0.42			
Fixed Effects	*Estimate*	*SE*	*df*	*t*	*p*
(Intercept)	0.44	0.34	279	1.30	0.193
**object happiness**	**0.50**	**0.05**	**825**	**9.94**	**< 0.001**
**subject sadness**	**0.39**	**0.11**	**765**	**3.45**	**< 0.001**
**object sadness**	**0.58**	**0.06**	**835**	**10.06**	**< 0.001**
**subject happiness**	**0.19**	**0.08**	**540**	**2.30**	**0.022**
**object happiness:subject sadness**	**-0.07**	**0.03**	**802**	**-2.57**	**0.010**
**object sadness:subject happiness**	**-0.05**	**0.02**	**819**	**-2.59**	**0.010**

Table 1. Linear mixed-effects model for prolonged (20 sec) songs ($N = 35$). The model explains 65% of the variance in the data. Bold values indicate statistical significance.

Brief (2 sec) songs

Random Effects	*Variance*	*SD*			
Participant	0.46	0.68			
Stimulus	0.11	0.33			
Fixed Effects	*Estimate*	*SE*	*df*	*t*	*p*
**(Intercept)**	**1.08**	**0.20**	**141**	**5.56**	**< 0.001**
**object happiness**	**0.47**	**0.03**	**794**	**16.88**	**< 0.001**
**object sadness**	**0.43**	**0.03**	**710**	**14.61**	**< 0.001**

Table 2. Linear mixed-effects model for brief (2 sec) songs ($N = 34$). The model explains 61% of the variance in the data. Bold values indicate statistical significance.

Prolonged (20 sec) images

Random Effects	*Variance*	*SD*			
Participant	0.29	0.54			
Stimulus	0.18	0.43			
Fixed Effects	*Estimate*	*SE*	*df*	*t*	*p*

**(Intercept)**	**2.52**	**0.21**	**174**	**11.98**	**< 0.001**
**object happiness**	**0.58**	**0.03**	**700**	**18.37**	**< 0.001**
**object sadness**	**0.11**	**0.03**	**657**	**3.46**	**< 0.001**

Table 3. Linear mixed-effects model for prolonged (20 sec) images (*N* = 33). The model explains 53% of the variance in the data. Bold values indicate statistical significance.

Brief (2 sec) images

Random Effects	*Variance*	*SD*			
Participant	0.77	0.88			
Stimulus	0.32	0.56			
Fixed Effects	*Estimate*	*SE*	*df*	*t*	*p*
**(Intercept)**	**3.76**	**0.45**	**504**	**8.40**	**< 0.001**
**object happiness**	**0.40**	**0.08**	**851**	**4.98**	**< 0.001**
**subject happiness**	**-0.21**	**0.10**	**836**	**-2.07**	**0.039**
object sadness	-0.16	0.08	851	-1.89	0.059
subject sadness	-0.27	0.17	846	-1.62	0.107
**object happiness:subject happiness**	**0.04**	**0.02**	**835**	**2.17**	**0.030**
**object sadness:subject sadness**	**0.06**	**0.03**	**835**	**2.10**	**0.036**
object happiness:subject sadness	0.03	0.03	836	1.26	0.209
**object sadness:subject happiness**	**0.04**	**0.02**	**830**	**2.17**	**0.030**

Table 4. Linear mixed-effects model for brief (2 sec) images (*N* = 36). The model explains 67% of the variance in the data. Bold values indicate statistical significance.

Linear mixed-effects models that include liking in place of beauty

Prolonged (20 sec) songs

Random Effects	*Variance*	*SD*			
Participant	0.49	0.70			
Stimulus	0.08	0.29			
Fixed Effects	*Estimate*	*SE*	*df*	*t*	*p*
(Intercept)	1.00	0.52	540	1.92	0.056
**object happiness**	**0.70**	**0.10**	**811**	**7.42**	**< 0.001**
subject happiness	-0.07	0.12	722	-0.58	0.565
**object sadness**	**0.35**	**0.10**	**810**	**3.54**	**< 0.001**
subject sadness	0.24	0.22	755	1.08	0.282
object happiness:subject happiness	0.00	0.02	820	-0.09	0.932
object sadness:subject sadness	0.00	0.04	779	-0.06	0.954
object happiness:subject sadness	-0.06	0.04	810	-1.60	0.109
object sadness:subject happiness	-0.01	0.02	812	-0.44	0.663

Table 5. Linear mixed-effects model for prolonged (20 sec) songs ($N = 35$). The model explains 61% of the variance in the data. Bold values indicate statistical significance.

Brief (2 sec) songs

Random Effects	*Variance*	*SD*			
Participant	0.61	0.78			
Stimulus	0.01	0.12			
Fixed Effects	*Estimate*	*SE*	*df*	*t*	*p*
(Intercept)	0.65	0.52	571	1.25	0.211
**object happiness**	**0.51**	**0.10**	**813**	**5.37**	**< 0.001**
subject happiness	0.14	0.15	799	0.91	0.363
**object sadness**	**0.42**	**0.10**	**810**	**4.29**	**< 0.001**
subject sadness	0.05	0.18	734	0.25	0.801
object happiness:subject happiness	0.00	0.03	812	0.17	0.865
object sadness:subject sadness	-0.02	0.03	811	-0.78	0.434
object happiness:subject sadness	0.03	0.03	808	1.08	0.281

object sadness:subject happiness	-0.01	0.03	810	-0.48	0.630

Table 6. Linear mixed-effects model for brief (2 sec) songs ($N = 34$). The model explains 60% of the variance in the data. Bold values indicate statistical significance.

Prolonged (20 sec) images

Random Effects	*Variance*	*SD*			
Participant	0.34	0.58			
Stimulus	0.22	0.47			
Fixed Effects	*Estimate*	*SE*	*df*	*t*	*p*
**(Intercept)**	**2.20**	**0.64**	**560**	**3.42**	**< 0.001**
**object happiness**	**0.53**	**0.10**	**762**	**5.15**	**< 0.001**
subject happiness	0.02	0.18	620	0.10	0.925
**object sadness**	**0.28**	**0.10**	**756**	**2.73**	**0.006**
subject sadness	0.22	0.20	564	1.10	0.271
object happiness:subject happiness	0.01	0.03	763	0.48	0.631
**object sadness:subject sadness**	**-0.09**	**0.03**	**766**	**-2.67**	**0.008**
object happiness:subject sadness	0.00	0.03	760	-0.01	0.990
object sadness:subject happiness	-0.04	0.03	746	-1.21	0.225

Table 7. Linear mixed-effects model for prolonged (20 sec) images ($N = 33$). The model explains 55% of the variance in the data. Bold values indicate statistical significance.

Brief (2 sec) images

Random Effects	*Variance*	*SD*			
Participant	0.56	0.75			
Stimulus	0.31	0.56			
Fixed Effects	*Estimate*	*SE*	*df*	*t*	*p*
**(Intercept)**	**4.05**	**0.47**	**501**	**8.66**	**< 0.001**
**object happiness**	**0.44**	**0.09**	**851**	**5.10**	**< 0.001**
**subject happiness**	**-0.25**	**0.11**	**808**	**-2.28**	**0.023**
**object sadness**	**-0.26**	**0.09**	**859**	**-2.90**	**0.004**
subject sadness	-0.29	0.18	836	-1.63	0.103
object happiness:subject happiness	0.02	0.02	842	1.11	0.266

**object sadness:subject sadness**	**0.08**	**0.03**	**844**	**2.79**	**0.005**
object happiness:subject sadness	0.01	0.03	845	0.49	0.625
**object sadness:subject happiness**	**0.07**	**0.02**	**838**	**3.54**	**< 0.001**

Table 8. Linear mixed-effects model for brief (2 sec) images (N = 36). The model explains 58% of the variance in the data. Bold values indicate statistical significance.

Linear mixed-effects models that include familiarity

Prolonged (20 sec) songs

Random Effects	*Variance*	*SD*			
Participant	0.68	0.83			
Stimulus	0.17	0.41			
Fixed Effects	*Estimate*	*SE*	*df*	*t*	*p*
(Intercept)	0.27	0.50	586	0.54	0.591
**object happiness**	**0.37**	**0.09**	**824**	**4.18**	**< 0.001**
subject happiness	0.11	0.11	788	0.98	0.326
**object sadness**	**0.53**	**0.09**	**826**	**5.72**	**< 0.001**
subject sadness	0.38	0.21	809	1.84	0.067
**familgen**	**0.14**	**0.04**	**710**	**3.85**	**< 0.001**
**familpar**	**0.15**	**0.04**	**825**	**4.04**	**< 0.001**
object happiness:subject happiness	0.01	0.02	821	0.63	0.530
object sadness:subject sadness	-0.01	0.04	818	-0.34	0.731
object happiness:subject sadness	-0.05	0.03	822	-1.55	0.122
object sadness:subject happiness	-0.04	0.02	820	-1.90	0.058

Table 9. Linear mixed-effects model for prolonged (20 sec) songs ($N = 35$). The model explains 67% of the variance in the data. Bold values indicate statistical significance.

Brief (2 sec) songs

Random Effects	*Variance*	*SD*			
Participant	0.35	0.59			
Stimulus	0.12	0.34			
Fixed Effects	*Estimate*	*SE*	*df*	*t*	*p*
(Intercept)	0.33	0.46	534	0.71	0.475
**object happiness**	**0.46**	**0.08**	**794**	**5.48**	**< 0.001**
subject happiness	0.11	0.13	754	0.85	0.396
**object sadness**	**0.39**	**0.09**	**786**	**4.49**	**< 0.001**
subject sadness	-0.01	0.16	651	-0.09	0.932
**familgen**	**0.23**	**0.03**	**713**	**7.44**	**< 0.001**

familpar	0.04	0.03	772	1.32	0.186
object happiness:subject happiness	-0.02	0.02	789	-0.80	0.426
object sadness:subject sadness	0.01	0.03	787	0.19	0.851
object happiness:subject sadness	-0.01	0.03	774	-0.40	0.690
object sadness:subject happiness	-0.01	0.02	792	-0.50	0.618

Table 10. Linear mixed-effects model for brief (2 sec) songs ($N = 34$). The model explains 65% of the variance in the data. Bold values indicate statistical significance.

Prolonged (20 sec) images

Random Effects	*Variance*	*SD*			
Participant	0.27	0.52			
Stimulus	0.16	0.40			
Fixed Effects	*Estimate*	*SE*	*df*	*t*	*p*
**(Intercept)**	**2.73**	**0.63**	**505**	**4.30**	**< 0.001**
**object happiness**	**0.35**	**0.10**	**750**	**3.50**	**< 0.001**
subject happiness	-0.19	0.18	583	-1.07	0.287
**object sadness**	**0.22**	**0.10**	**740**	**2.16**	**0.031**
subject sadness	-0.08	0.20	510	-0.41	0.683
**familgen**	**0.12**	**0.03**	**714**	**3.76**	**< 0.001**
familpar	-0.04	0.05	624	-0.86	0.391
object happiness:subject happiness	0.05	0.03	755	1.83	0.068
object sadness:subject sadness	-0.05	0.03	757	-1.51	0.133
object happiness:subject sadness	0.05	0.03	748	1.48	0.139
object sadness:subject happiness	0.00	0.03	731	-0.15	0.883

Table 11. Linear mixed-effects model for prolonged (20 sec) images ($N = 33$). The model explains 54% of the variance in the data. Bold values indicate statistical significance.

Brief (2 sec) images

Random Effects	*Variance*	*SD*			
Participant	0.73	0.86			
Stimulus	0.28	0.53			
Fixed Effects	*Estimate*	*SE*	*df*	*t*	*p*

**(Intercept)**	**3.37**	**0.45**	**533**	**7.47**	**< 0.001**
**object happiness**	**0.37**	**0.08**	**851**	**4.66**	**< 0.001**
**subject happiness**	**-0.22**	**0.10**	**834**	**-2.15**	**0.032**
object sadness	-0.15	0.08	852	-1.88	0.060
subject sadness	-0.26	0.16	846	-1.56	0.119
**familgen**	**0.14**	**0.03**	**838**	**4.71**	**< 0.001**
familpar	-0.04	0.04	813	-1.00	0.319
**object happiness:subject happiness**	**0.04**	**0.02**	**835**	**2.31**	**0.021**
**object sadness:subject sadness**	**0.06**	**0.03**	**835**	**2.04**	**0.042**
object happiness:subject sadness	0.03	0.03	836	1.15	0.251
**object sadness:subject happiness**	**0.04**	**0.02**	**830**	**2.21**	**0.027**

Table 12. Linear mixed-effects model for brief (2 sec) images ($N = 36$). The model explains 67% of the variance in the data. Bold values indicate statistical significance.

Linear mixed-effects models using PANAS positive and negative affect scores in place of object happiness and object sadness ratings

Prolonged (20 sec) songs

Random Effects	*Variance*	*SD*			
Participant	0.66	0.82			
Stimulus	0.17	0.41			
Fixed Effects	*Estimate*	*SE*	*df*	*t*	*p*
(Intercept)	0.61	0.78	630	0.78	0.436
**object happiness**	**0.36**	**0.12**	**827**	**3.01**	**0.003**
PANASpos	0.02	0.02	658	0.99	0.324
**object sadness**	**0.73**	**0.13**	**820**	**5.73**	**< 0.001**
PANASneg	0.04	0.06	809	0.74	0.457
object happiness:PANASpos	0.00	0.00	827	1.74	0.083
object sadness:PANASneg	0.00	0.01	806	0.13	0.897
object happiness:PANASneg	-0.01	0.01	819	-0.83	0.408
**PANASpos:object sadness**	**-0.01**	**0.00**	**813**	**-4.15**	**< 0.001**

Table 13. Linear mixed-effects model for prolonged (20 sec) songs ($N = 35$). The model explains 65% of the variance in the data. Bold values indicate statistical significance.

Brief (2 sec) songs

Random Effects	*Variance*	*SD*			
Participant	0.44	0.66			
Stimulus	0.11	0.33			
Fixed Effects	*Estimate*	*SE*	*df*	*t*	*p*
**(Intercept)**	**1.18**	**0.60**	**446**	**1.97**	**0.049**
**object happiness**	**0.28**	**0.11**	**733**	**2.56**	**0.011**
PANASpos	-0.01	0.02	774	-0.54	0.590
**object sadness**	**0.31**	**0.10**	**773**	**3.03**	**0.003**
PANASneg	0.01	0.03	461	0.33	0.745
object happiness:PANASpos	0.00	0.00	779	1.58	0.115
object sadness:PANASneg	0.00	0.00	790	-0.20	0.846

object happiness:PANASneg	0.01	0.00	720	1.33	0.184
PANASpos:object sadness	0.01	0.00	791	1.82	0.069

Table 14. Linear mixed-effects model for brief (2 sec) songs (*N* = 34). The model explains 61% of the variance in the data. Bold values indicate statistical significance.

Prolonged (20 sec) images

Random Effects	*Variance*	*SD*			
Participant	0.28	0.53			
Stimulus	0.17	0.42			
Fixed Effects	*Estimate*	*SE*	*df*	*t*	*p*
**(Intercept)**	**1.36**	**0.69**	**423**	**1.98**	**0.048**
**object happiness**	**0.65**	**0.12**	**725**	**5.55**	**< 0.001**
PANASpos	0.02	0.02	575	1.12	0.262
**object sadness**	**0.53**	**0.12**	**724**	**4.59**	**< 0.001**
PANASneg	0.04	0.03	643	1.37	0.171
object happiness:PANASpos	0.00	0.00	721	-0.38	0.707
**object sadness:PANASneg**	**-0.01**	**0.01**	**773**	**-2.52**	**0.012**
object happiness:PANASneg	0.00	0.01	767	-0.60	0.552
**PANASpos:object sadness**	**-0.01**	**0.00**	**703**	**-2.68**	**0.008**

Table 15. Linear mixed-effects model for prolonged (20 sec) images (*N* = 33). The model explains 54% of the variance in the data. Bold values indicate statistical significance.

Brief (2 sec) images

Random Effects	*Variance*	*SD*			
Participant	0.74	0.86			
Stimulus	0.34	0.58			
Fixed Effects	*Estimate*	*SE*	*df*	*t*	*p*
**(Intercept)**	**4.57**	**0.66**	**412**	**6.90**	**< 0.001**
object happiness	0.14	0.11	854	1.29	0.197
PANASpos	-0.02	0.02	751	-1.07	0.283
object sadness	-0.05	0.11	847	-0.45	0.652
**PANASneg**	**-0.10**	**0.04**	**668**	**-2.64**	**0.009**

**object happiness:PANASpos**	**0.01**	**0.00**	**840**	**2.23**	**0.026**
object sadness:PANASneg	0.01	0.01	840	1.04	0.300
**object happiness:PANASneg**	**0.02**	**0.01**	**841**	**3.60**	**< 0.001**
PANASpos:object sadness	0.00	0.00	834	0.31	0.757

Table 16. Linear mixed-effects model for brief (2 sec) images ($N = 36$). The model explains 67% of the variance in the data. Bold values indicate statistical significance.

Linear mixed-effects models that include AReA or BMRQ and art or music training

Prolonged (20 sec) songs

Random Effects	*Variance*	*SD*			
Participant	0.58	0.76			
Stimulus	0.18	0.42			
Fixed Effects	*Estimate*	*SE*	*df*	*t*	*p*
(Intercept)	-0.364	1.025	52	-0.355	0.724
**object happiness**	**0.475**	**0.089**	**821**	**5.309**	**< 0.001**
subject happiness	0.139	0.111	786	1.252	0.211
**object sadness**	**0.615**	**0.094**	**824**	**6.545**	**< 0.001**
**subject sadness**	**0.488**	**0.213**	**803**	**2.293**	**0.022**
BMRQ	0.011	0.012	38	0.896	0.376
music_training_years	-0.016	0.028	38	-0.569	0.573
object happiness:subject happiness	0.013	0.020	820	0.639	0.523
object sadness:subject sadness	-0.025	0.036	811	-0.704	0.481
**object happiness:subject sadness**	**-0.075**	**0.035**	**820**	**-2.155**	**0.032**
**object sadness:subject happiness**	**-0.049**	**0.022**	**818**	**-2.244**	**0.025**

Table 17. Linear mixed-effects model for prolonged (20 sec) songs ($N = 35$). The model explains 65% of the variance in the data. Bold values indicate statistical significance.

Brief (2 sec) songs

Random Effects	*Variance*	*SD*			
Participant	0.39	0.62			
Stimulus	0.11	0.33			
Fixed Effects	*Estimate*	*SE*	*df*	*t*	*p*
(Intercept)	-0.31	0.80	49	-0.38	0.703
**object happiness**	**0.46**	**0.09**	**800**	**5.20**	**< 0.001**
subject happiness	-0.03	0.14	776	-0.23	0.816
**object sadness**	**0.38**	**0.09**	**794**	**4.17**	**< 0.001**
subject sadness	-0.08	0.16	694	-0.48	0.632
**BMRQ**	**0.02**	**0.01**	**36**	**2.28**	**0.029**

music_training_years	-0.01	0.03	34	-0.50	0.620
object happiness:subject happiness	0.00	0.02	794	0.16	0.874
object sadness:subject sadness	0.01	0.03	790	0.45	0.652
object happiness:subject sadness	0.00	0.03	782	-0.01	0.990
object sadness:subject happiness	0.01	0.02	795	0.43	0.665

Table 18. Linear mixed-effects model for brief (2 sec) songs (*N* = 34). The model explains 62% of the variance in the data. Bold values indicate statistical significance.

Prolonged (20 sec) images

Random Effects	*Variance*	*SD*			
Participant	0.25	0.50			
Stimulus	0.19	0.44			
Fixed Effects	*Estimate*	*SE*	*df*	*t*	*p*
**(Intercept)**	**2.78**	**0.80**	**134**	**3.47**	**< 0.001**
**object happiness**	**0.39**	**0.10**	**744**	**3.89**	**< 0.001**
subject happiness	-0.12	0.18	564	-0.66	0.509
**object sadness**	**0.21**	**0.10**	**737**	**2.12**	**0.034**
subject sadness	-0.08	0.20	503	-0.41	0.685
AREA	0.00	0.01	33	0.15	0.881
art_training_years	0.03	0.02	34	1.85	0.074
object happiness:subject happiness	0.04	0.03	747	1.52	0.128
object sadness:subject sadness	-0.05	0.03	767	-1.42	0.158
object happiness:subject sadness	0.05	0.03	755	1.38	0.167
object sadness:subject happiness	-0.01	0.03	718	-0.40	0.686

Table 19. Linear mixed-effects model for prolonged (20 sec) images (*N* = 33). The model explains 54% of the variance in the data. Bold values indicate statistical significance.

Brief (2 sec) images

Random Effects	*Variance*	*SD*			
Participant	0.64	0.80			
Stimulus	0.32	0.57			
Fixed Effects	*Estimate*	*SE*	*df*	*t*	*p*

**(Intercept)**	**2.29**	**0.73**	**65**	**3.12**	**0.003**
**object happiness**	**0.39**	**0.08**	**852**	**4.90**	**< 0.001**
**subject happiness**	**-0.22**	**0.10**	**830**	**-2.10**	**0.036**
**object sadness**	**-0.16**	**0.08**	**853**	**-1.96**	**0.050**
subject sadness	-0.26	0.16	844	-1.60	0.110
**AREA**	**0.04**	**0.02**	**37**	**2.35**	**0.024**
art_training_years	0.00	0.04	37	-0.13	0.900
**object happiness:subject happiness**	**0.04**	**0.02**	**838**	**2.16**	**0.031**
**object sadness:subject sadness**	**0.06**	**0.03**	**838**	**2.11**	**0.035**
object happiness:subject sadness	0.03	0.03	839	1.27	0.205
**object sadness:subject happiness**	**0.04**	**0.02**	**833**	**2.16**	**0.031**

Table 20. Linear mixed-effects model for brief (2 sec) images (*N* = 36). The model explains 68% of the variance in the data. Bold values indicate statistical significance.

Linear mixed-effects models that include QCAE

Prolonged (20 sec) songs

Random Effects	*Variance*	*SD*			
Participant	0.60	0.77			
Stimulus	0.18	0.42			
Fixed Effects	*Estimate*	*SE*	*df*	*t*	*p*
(Intercept)	0.36	1.02	66	0.35	0.724
**object happiness**	**0.47**	**0.09**	**821**	**5.30**	**< 0.001**
subject happiness	0.15	0.11	786	1.38	0.167
**object sadness**	**0.62**	**0.09**	**824**	**6.55**	**< 0.001**
**subject sadness**	**0.50**	**0.21**	**798**	**2.34**	**0.019**
QCAE	0.00	0.01	39	0.01	0.992
object happiness:subject happiness	0.01	0.02	821	0.68	0.498
object sadness:subject sadness	-0.03	0.04	815	-0.70	0.482
**object happiness:subject sadness**	**-0.07**	**0.04**	**821**	**-2.15**	**0.032**
**object sadness:subject happiness**	**-0.05**	**0.02**	**818**	**-2.21**	**0.027**

Table 21. Linear mixed-effects model for prolonged (20 sec) songs ($N = 35$). The model explains 64% of the variance in the data. Bold values indicate statistical significance.

Brief (2 sec) songs

Random Effects	*Variance*	*SD*			
Participant	0.33	0.57			
Stimulus	0.11	0.34			
Fixed Effects	*Estimate*	*SE*	*df*	*t*	*p*
(Intercept)	-1.01	0.78	55	-1.30	0.199
**object happiness**	**0.46**	**0.09**	**790**	**5.22**	**< 0.001**
subject happiness	-0.06	0.14	768	-0.42	0.675
**object sadness**	**0.37**	**0.09**	**783**	**4.07**	**< 0.001**
subject sadness	-0.03	0.16	616	-0.19	0.847
**QCAE**	**0.02**	**0.01**	**37**	**3.45**	**0.001**
object happiness:subject happiness	0.01	0.02	790	0.35	0.724

object sadness:subject sadness	0.01	0.03	779	0.32	0.751
object happiness:subject sadness	-0.01	0.03	765	-0.29	0.774
object sadness:subject happiness	0.01	0.02	793	0.60	0.551

Table 22. Linear mixed-effects model for brief (2 sec) songs (*N* = 34). The model explains 64% of the variance in the data. Bold values indicate statistical significance.

Prolonged (20 sec) images

Random Effects	*Variance*	*SD*			
Participant	0.77	0.88			
Stimulus	0.25	0.50			
Fixed Effects	*Estimate*	*SE*	*df*	*t*	*p*
**(Intercept)**	**2.27**	**1.10**	**55**	**2.07**	**0.043**
**object happiness**	**0.41**	**0.09**	**826**	**4.38**	**< 0.001**
subject happiness	-0.09	0.12	787	-0.73	0.463
object sadness	-0.03	0.09	835	-0.29	0.771
subject sadness	-0.14	0.19	777	-0.76	0.450
QCAE	0.01	0.01	35	1.13	0.267
object happiness:subject happiness	0.04	0.02	816	1.73	0.083
object sadness:subject sadness	0.02	0.03	822	0.54	0.593
object happiness:subject sadness	0.01	0.03	822	0.39	0.699
object sadness:subject happiness	0.02	0.02	820	0.99	0.324

Table 23. Linear mixed-effects model for prolonged (20 sec) images (*N* = 33). The model explains 61% of the variance in the data. Bold values indicate statistical significance.

Brief (2 sec) images

Random Effects	*Variance*	*SD*			
Participant	0.24	0.49			
Stimulus	0.25	0.50			
Fixed Effects	*Estimate*	*SE*	*df*	*t*	*p*
**(Intercept)**	**2.65**	**0.71**	**74**	**3.73**	**< 0.001**
**object happiness**	**0.28**	**0.08**	**758**	**3.33**	**< 0.001**
**subject happiness**	**-0.41**	**0.14**	**735**	**-2.93**	**0.004**

object sadness	0.02	0.09	792	0.22	0.827
**subject sadness**	**-0.35**	**0.17**	**703**	**-2.03**	**0.043**
**QCAE**	**0.01**	**0.00**	**34**	**2.54**	**0.016**
**object happiness:subject happiness**	**0.07**	**0.02**	**787**	**3.43**	**< 0.001**
object sadness:subject sadness	0.01	0.03	799	0.40	0.688
**object happiness:subject sadness**	**0.07**	**0.03**	**794**	**2.68**	**0.008**
object sadness:subject happiness	0.03	0.02	796	1.17	0.244

Table 24. Linear mixed-effects model for brief (2 sec) images ($N = 36$). The model explains 61% of the variance in the data. Bold values indicate statistical significance.

Linear mixed-effects models that only include data from participants with above-sample-mean QCAE scores

Prolonged (20 sec) songs

Random Effects	*Variance*	*SD*			
Participant	0.23	0.48			
Stimulus	0.54	0.74			
Fixed Effects	*Estimate*	*SE*	*df*	*t*	*p*
(Intercept)	-0.23	0.75	279	-0.31	0.760
**object happiness**	**0.52**	**0.12**	**456**	**4.40**	**< 0.001**
**subject happiness**	**0.56**	**0.20**	**282**	**2.79**	**0.006**
**object sadness**	**0.65**	**0.13**	**460**	**5.14**	**< 0.001**
**subject sadness**	**0.67**	**0.31**	**462**	**2.20**	**0.028**
object happiness:subject happiness	-0.04	0.03	459	-1.22	0.225
object sadness:subject sadness	-0.04	0.05	462	-0.84	0.401
object happiness:subject sadness	-0.08	0.05	464	-1.64	0.101
**object sadness:subject happiness**	**-0.10**	**0.03**	**457**	**-3.18**	**0.002**

Table 25. Linear mixed-effects model for prolonged (20 sec) songs ($N = 20$). The model explains 62% of the variance in the data. Bold values indicate statistical significance.

Brief (2 sec) songs

Random Effects	*Variance*	*SD*			
Participant	0.25	0.50			
Stimulus	0.35	0.59			
Fixed Effects	*Estimate*	*SE*	*df*	*t*	*p*
(Intercept)	1.34	0.78	437	1.72	0.086
**object happiness**	**0.39**	**0.13**	**433**	**3.15**	**0.002**
subject happiness	0.14	0.22	434	0.66	0.508
**object sadness**	**0.47**	**0.13**	**442**	**3.80**	**< 0.001**
subject sadness	-0.24	0.23	434	-1.07	0.286
object happiness:subject happiness	0.00	0.03	429	-0.10	0.923
object sadness:subject sadness	0.03	0.04	430	0.94	0.346

object happiness:subject sadness	0.04	0.04	432	1.18	0.239
object sadness:subject happiness	-0.03	0.03	430	-0.95	0.345

Table 26. Linear mixed-effects model for brief (2 sec) songs (*N* = 19). The model explains 56% of the variance in the data. Bold values indicate statistical significance.

Prolonged (20 sec) images

Random Effects	*Variance*	*SD*			
Participant	0.26	0.51			
Stimulus	0.24	0.49			
Fixed Effects	*Estimate*	*SE*	*df*	*t*	*p*
(Intercept)	1.50	1.03	267	1.45	0.147
**object happiness**	**0.46**	**0.16**	**385**	**2.83**	**0.005**
subject happiness	0.50	0.31	361	1.60	0.110
**object sadness**	**0.50**	**0.16**	**389**	**3.09**	**0.002**
subject sadness	0.03	0.33	359	0.08	0.939
object happiness:subject happiness	0.00	0.05	387	-0.01	0.990
object sadness:subject sadness	-0.04	0.05	394	-0.76	0.450
object happiness:subject sadness	0.05	0.05	390	1.04	0.299
**object sadness:subject happiness**	**-0.13**	**0.05**	**389**	**-2.80**	**0.005**

Table 27. Linear mixed-effects model for prolonged (20 sec) images (*N* = 20). The model explains 59% of the variance in the data. Bold values indicate statistical significance.

Brief (2 sec) images

Random Effects	*Variance*	*SD*			
Participant	0.42	0.65			
Stimulus	0.32	0.56			
Fixed Effects	*Estimate*	*SE*	*df*	*t*	*p*
**(Intercept)**	**4.49**	**0.54**	**339**	**8.29**	**< 0.001**
**object happiness**	**0.31**	**0.10**	**515**	**3.09**	**0.002**
**subject happiness**	**-0.46**	**0.14**	**460**	**-3.36**	**< 0.001**
object sadness	-0.13	0.10	519	-1.27	0.203
subject sadness	-0.19	0.19	508	-0.97	0.331

**object happiness:subject happiness**	**0.07**	**0.02**	**506**	**3.06**	**0.002**
object sadness:subject sadness	0.04	0.03	504	1.18	0.239
object happiness:subject sadness	0.03	0.03	503	0.79	0.430
object sadness:subject happiness	0.05	0.02	503	1.95	0.052

Table 28. Linear mixed-effects model for brief (2 sec) images ($N = 19$). The model explains 59% of the variance in the data. Bold values indicate statistical significance.

Linear mixed-effects models that only include data from participants with below-sample-mean QCAE scores

Prolonged (20 sec) songs

Random Effects	*Variance*	*SD*			
Participant	0.09	0.30			
Stimulus	0.85	0.92			
Fixed Effects	*Estimate*	*SE*	*df*	*t*	*p*
(Intercept)	0.77	0.72	249	1.07	0.288
**object happiness**	**0.40**	**0.14**	**351**	**2.87**	**0.004**
subject happiness	-0.05	0.14	354	-0.35	0.727
**object sadness**	**0.62**	**0.15**	**352**	**4.10**	**< 0.001**
subject sadness	0.24	0.32	354	0.77	0.443
object happiness:subject happiness	0.05	0.03	348	1.77	0.078
object sadness:subject sadness	0.03	0.07	351	0.38	0.707
object happiness:subject sadness	-0.09	0.06	347	-1.57	0.117
object sadness:subject happiness	-0.02	0.03	343	-0.49	0.626

Table 29. Linear mixed-effects model for prolonged (20 sec) songs ($N = 15$). The model explains 70% of the variance in the data. Bold values indicate statistical significance.

Brief (2 sec) songs

Random Effects	*Variance*	*SD*			
Participant	0.04	0.20			
Stimulus	0.35	0.59			
Fixed Effects	*Estimate*	*SE*	*df*	*t*	*p*
**(Intercept)**	**1.53**	**0.60**	**113**	**2.54**	**0.013**
**object happiness**	**0.33**	**0.17**	**350**	**2.01**	**0.045**
subject happiness	-0.19	0.18	326	-1.08	0.281
**object sadness**	**0.55**	**0.17**	**355**	**3.23**	**0.001**
subject sadness	-0.10	0.23	93	-0.43	0.668
object happiness:subject happiness	0.06	0.05	348	1.17	0.243
**object sadness:subject sadness**	**-0.17**	**0.06**	**344**	**-2.98**	**0.003**

object happiness:subject sadness	0.03	0.06	348	0.47	0.637
object sadness:subject happiness	0.02	0.05	353	0.34	0.731

Table 30. Linear mixed-effects model for brief (2 sec) songs (*N* = 15). The model explains 66% of the variance in the data. Bold values indicate statistical significance.

Prolonged (20 sec) images

Random Effects	*Variance*	*SD*			
Participant	0.16	0.40			
Stimulus	0.85	0.92			
Fixed Effects	*Estimate*	*SE*	*df*	*t*	*p*
**(Intercept)**	**2.66**	**0.72**	**267**	**3.67**	**< 0.001**
**object happiness**	**0.36**	**0.13**	**419**	**2.68**	**0.008**
subject happiness	-0.01	0.16	418	-0.05	0.962
object sadness	0.12	0.13	424	0.89	0.376
subject sadness	-0.13	0.28	376	-0.46	0.647
object happiness:subject happiness	0.03	0.03	418	1.07	0.287
object sadness:subject sadness	-0.07	0.05	413	-1.37	0.173
object happiness:subject sadness	0.10	0.06	421	1.75	0.081
object sadness:subject happiness	0.04	0.03	421	1.32	0.187

Table 31. Linear mixed-effects model for prolonged (20 sec) images (*N* = 15). The model explains 57% of the variance in the data. Bold values indicate statistical significance.

Brief (2 sec) images

Random Effects	*Variance*	*SD*			
Participant	0.07	0.27			
Stimulus	0.21	0.46			
Fixed Effects	*Estimate*	*SE*	*df*	*t*	*p*
**(Intercept)**	**4.45**	**0.69**	**73**	**6.42**	**< 0.001**
**object happiness**	**0.28**	**0.14**	**245**	**1.97**	**0.050**
**subject happiness**	**-0.59**	**0.21**	**180**	**-2.82**	**0.005**
object sadness	0.15	0.15	274	0.95	0.341
**subject sadness**	**-0.56**	**0.27**	**74**	**-2.07**	**0.042**

**object happiness:subject happiness**	**0.08**	**0.04**	**263**	**2.00**	**0.046**
object sadness:subject sadness	-0.10	0.06	282	-1.74	0.083
object happiness:subject sadness	0.11	0.06	281	1.94	0.054
object sadness:subject happiness	0.01	0.05	279	0.30	0.766

Table 32. Linear mixed-effects model for brief (2 sec) images (*N* = 15). The model explains 73% of the variance in the data. Bold values indicate statistical significance.

Linear mixed-effects models looking at the effect of prior subject emotion on object emotion ratings

Random Effects	*Variance*	*SD*			
Participant	0.80	0.90			
Stimulus	0.94	0.97			
Fixed Effects	*Estimate*	*SE*	*df*	*t*	*p*
(Intercept)	2.99	0.23	237	12.95	< 0.001
subject happiness	0.05	0.04	1,809	1.14	0.26
subject sadness	0.01	0.05	2,520	0.13	0.90

Table 33. Linear mixed-effects model for object happiness ($N = 69$). The model explains 49% of the variance in the data.

Random Effects	*Variance*	*SD*			
Participant	0.75	0.86			
Stimulus	1.04	1.02			
Fixed Effects	*Estimate*	*SE*	*df*	*t*	*p*
(Intercept)	2.64	0.23	206	11.53	< 0.001
subject happiness	0.06	0.04	1,915	1.65	0.10
subject sadness	0.01	0.04	2,601	0.33	0.74

Table 34. Linear mixed-effects model for object sadness ($N = 69$). The model explains 53% of the variance in the data.

Count of participants broken out by stimulus category and mood induction type

Stimulus	Duration	Mood induction	Participant count
Songs	Prolonged (20 s)	Happy	12
		Sad	12
		Neutral	11
	Brief (2 s)	Happy	12
		Sad	10
		Neutral	12
Images	Prolonged (20 s)	Happy	12
		Sad	10
		Neutral	11
	Brief (2 s)	Happy	12
		Sad	12
		Neutral	12

Table 35. Count of participants broken out by stimulus category and mood induction type. Each participant rated stimuli from two stimulus categories (songs of one duration and images of one duration) and saw only one mood induction video.

Correlations between happiness and sadness of subjects, images, and songs

Factors	*r*	*p*
Subject happiness v. subject sadness	-0.27	< 0.001
Image happiness v. image sadness	-0.32	< 0.001
Song happiness v. song sadness	-0.15	< 0.001

Table 36. Pearson correlations between happiness and sadness of subjects, images, and songs. $N = 69$.

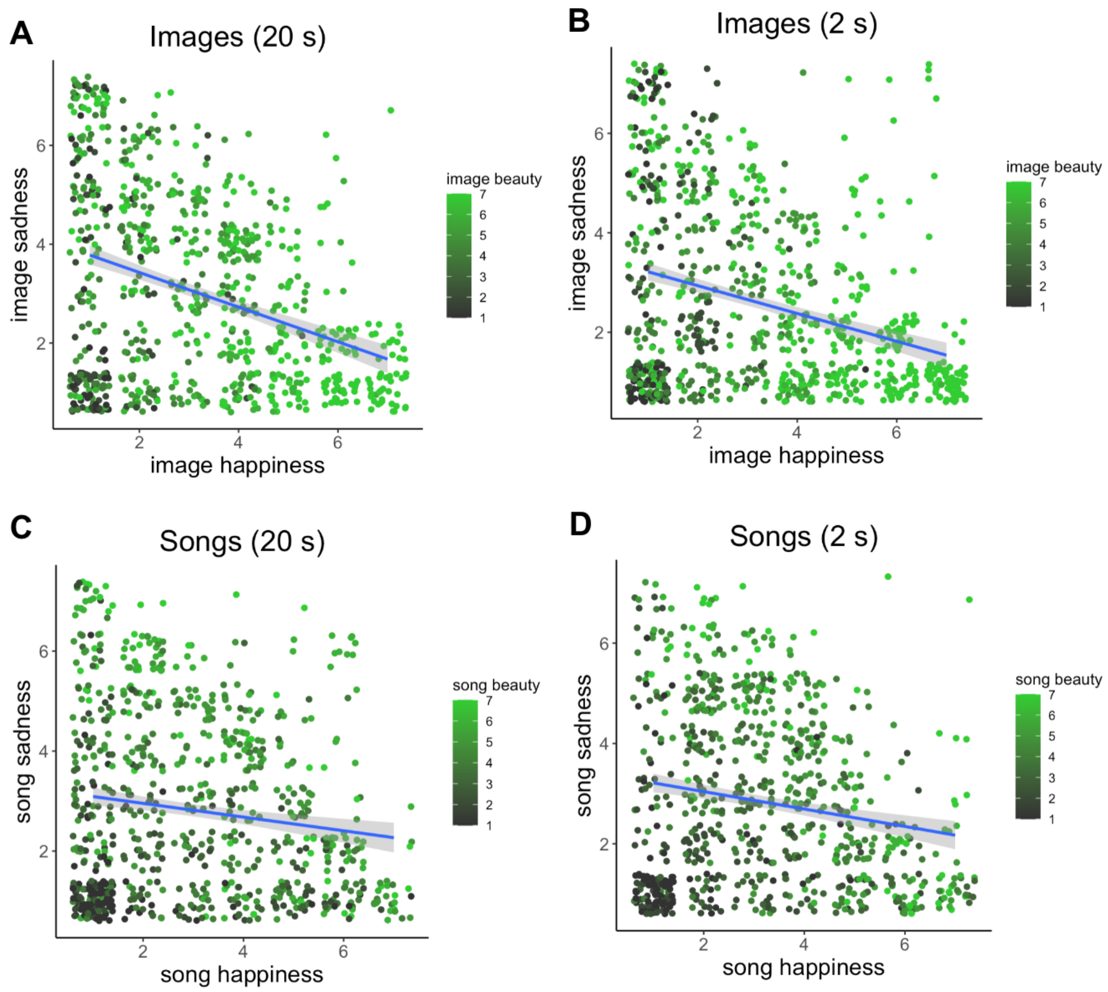

Figure 1. Scatterplots and regression lines showing the correlations between object happiness and sadness as well as associated beauty rating (color). $N = 33$ for Figure 1A, $N = 36$ for 1B, $N = 35$ for 1C, and $N = 34$ for 1D.

Barplots showing counts of subject happiness and sadness ratings, split by mood induction group

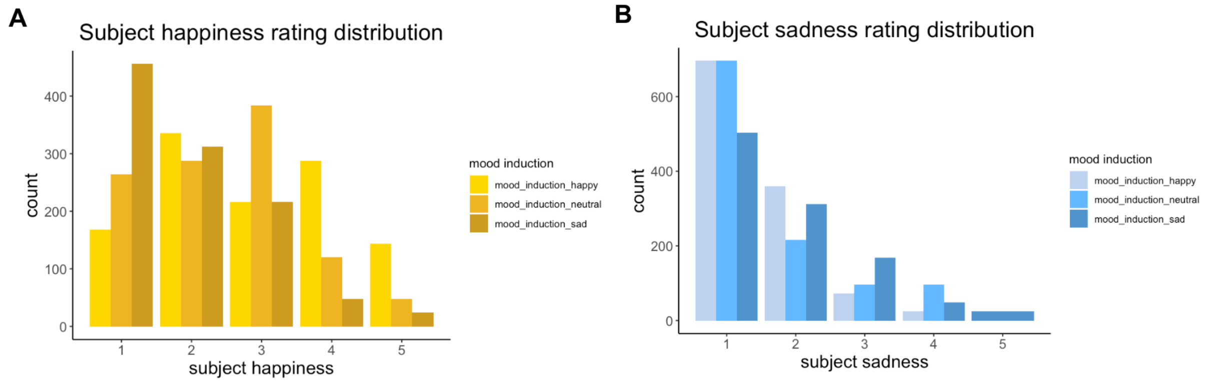

Figure 2. Barplots showing counts of participant happiness and sadness ratings on a 7-point Likert scale from "Not at all" to "Very," taken from the PANAS mood questionnaire (Watson, 1998). Plots include ratings for each stimulus (24 images and 24 songs) from all 69 included participants.

Barplots showing counts of image and song beauty, happiness, and sadness ratings

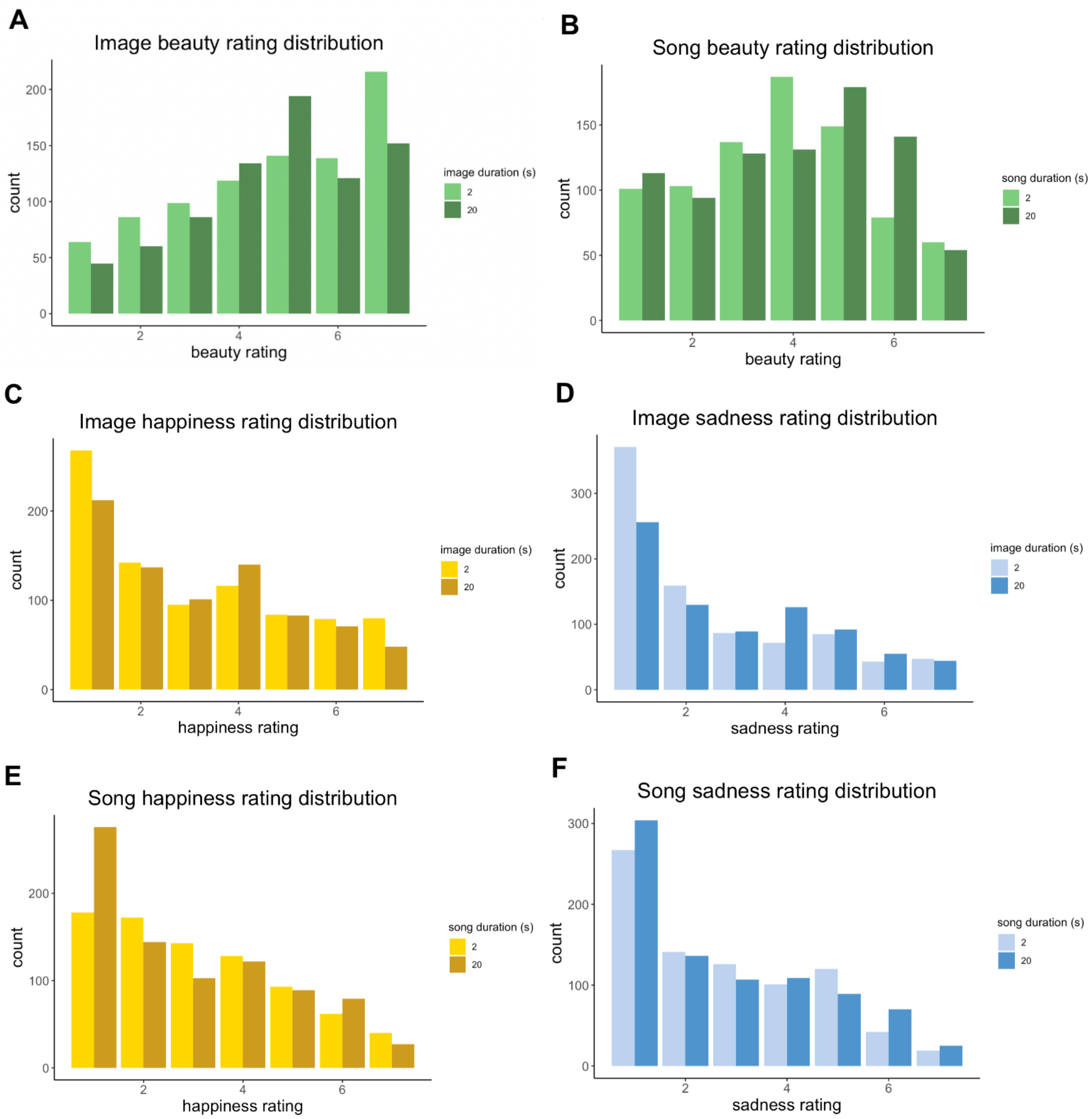

Figure 3. Barplots showing counts of image and song beauty, happiness, and sadness ratings on a 7-point Likert scale from "Not at all" to "Very." Plots include ratings for each stimulus (24 images and 24 songs) from all 69 included participants.
